# Assistive Technology and Supplementary Treatment for Individuals with Rett Syndrome

**DOI:** 10.1100/tsw.2007.5

**Published:** 2007-06-12

**Authors:** Meir Lotan

**Affiliations:** ^1^ Israeli Rett Center, National Evaluation Team, Chaim Sheba Medical Center, Tel HaShomer, Ramat Gan, Israel; ^2^ Department of Physical Therapy, Academic College of Judea and Samaria, Ariel, Israel

**Keywords:** Rett syndrome, assistive technology

## Abstract

Rett syndrome (RS) is a neurological disorder, affecting mainly females, caused by MECP2 mutations usually resulting in severe physical disability. Due to the physical challenges faced by the individual with RS and her family, her rehabilitation program should support her throughout different daily activities, contexts, and surroundings. Rehabilitation interventions to reverse physical impairments include exercise of various types and different physical modalities. Nevertheless, in the vast majority of cases, hands-on therapeutic intervention opportunities are available for the client through a minute part of her waking hours. Hence, a supplementary system is required in order to engulf the child with a comprehensive network of support. Supplementary intervention can support physical impairment by introducing adaptive techniques, environmental modifications, and assistive technologies. The therapy program of an individual with RS should include the use of assistive technology when such devices improve the user's performance. The term “supplementary management” relates to the fact that this intervention may be performed by nonprofessionals with the supervision of a qualified therapist. Such an intervention can further support the therapeutic goals of the child, at a time when direct intervention is not supplied. The present article will review the available literature on the topic of assistive technology, incorporating the clinical knowledge of the author in the field of RS.

## INTRODUCTION

Intervention for persons with Rett syndrome (RS) is aimed at compensation and reduction of physical impairment and includes therapeutic intervention, adaptive techniques, environmental modifications, and assistive technologies[1]. Para therapeutic disciplines, such as physiotherapy, occupational therapy, music therapy, or others, should not be limited to what is administered in the treatment room or during individually applied sessions (also known as direct therapy). In order to attain a continuous effect, therapists and caring staff should work as a team with the family of the individual with RS to construct a comprehensive intervention program that includes both the educational facility as well as the individual's residence. Kerr[2] has asserted that exercise taught to caregivers is highly recommended throughout the life of the individual with RS and she suggests that regular activity is essential for the long-lasting health of this population[2].

The aim of this article is to present the use of assistive technology in order to construct a support network around the individual with RS. ("Assistive technology" refers specifically to devices used to compensate for physical limitations. The term may be used narrowly to refer to special tools used to accomplish an activity [e.g., a walker], or it may include orthotics [e.g., braces, splints], as well as some adaptive techniques, which refer to modifications in the way an activity is carried out[1]. Such day-by-day and hour-to-hour support is advised, suggesting that everyone who is in contact with the individual with RS will be aware of her needs and supportive to her supplementary management program.

The term "supplementary management program" implies continuous care for the individual by use of time, intensity of appropriate handling, and suitable assistive devices. A supplementary management program will accompany and support the client's therapeutic regime throughout the day. Such a program can help the individual with RS during all the wearying battles she must constantly wage against the limitations that RS imposes on her. Since RS is constantly present and never relents, treatment management should continue through every available minute of every day. This article will present possibilities of interventions that might be used even during the resting hours of the individual with RS, as well as utilizing her play and recreation time for introducing therapeutic elements without harming the joy of the user and the quality of her life.

The scope of this article is broad with the intention to accommodate professionals as well as caregivers of individuals with RS and, therefore, on occasion, could be found written with too much detail by the experienced therapist. When assistive devices are warranted, it is important that equipment and aids are prepared and chosen with the help of a multi-professional team that centers on the client, the family, and other primary caregivers. Also involved in this process are the staff of the educational facility, agencies responsible for funding, and members of the community who are in contact with the family. All these people must each contribute their part to attain the best possible results. All these aspects constitute the full scope of the supplementary management program.

Despite widespread use of assistive technology[3] in recent years for various populations, research on assistive technology for the general user population as well as for individuals with RS is relatively sparse; therefore, the scope of this article is mainly clinical. While reviewing available literature on issues of assistive technology, this article will present ideas that have been found successful for individuals with RS and might be found useful for others bearing the same diagnosis and who are in need of assistive technology.

Due to individual expression of RS and due to the ever-changing abilities and challenges posed on this population, the therapist should not limit him\herself to conventions that have already been tried. When there are no existing solutions, there is need to use imagination, to explore some crazy notions, and to experiment with new ideas. In the words of Yalom[4] taken from another professional context, but with the same spirit in mind, "inspire yourself to create a new treatment for each client." Anything that might get the client with RS to improve her disposition or performance is worth investigating.

## BASIC ASPECTS OF ASSISTIVE TECHNOLOGY

It is common knowledge that there are many negative aspects associated with the acquisition and use of assistive technology[5,6,7,8]. The main reasons that clients (in our case, the individual with RS and her family) abandon assistive technology is that they feel the equipment does not meet their needs[9], the equipment is in disrepair or does not fit properly[10], or because they have difficulty operating or using it[11]. Therefore, and due to the complex nature of assistive technology and the ample factors associated with its purchase, it is advised that experienced paraprofessionals knowledgeable in the area of assistive technology and RS be involved in the process of adapting the most appropriate equipment. Furthermore, when choosing the appropriate assistive technology, the following factors should be taken into consideration:

- The child's physical profile (deformities, motor ability, and tendency towards pressure sores)
- Environmental limitations (elevators, transportation vehicles, access paths)
- Behavioral needs (such as self-inflicted injuries)
- Daily routine (numerous transitions vs. remaining in one location)
- Future needs (due to change of location or changes in function)

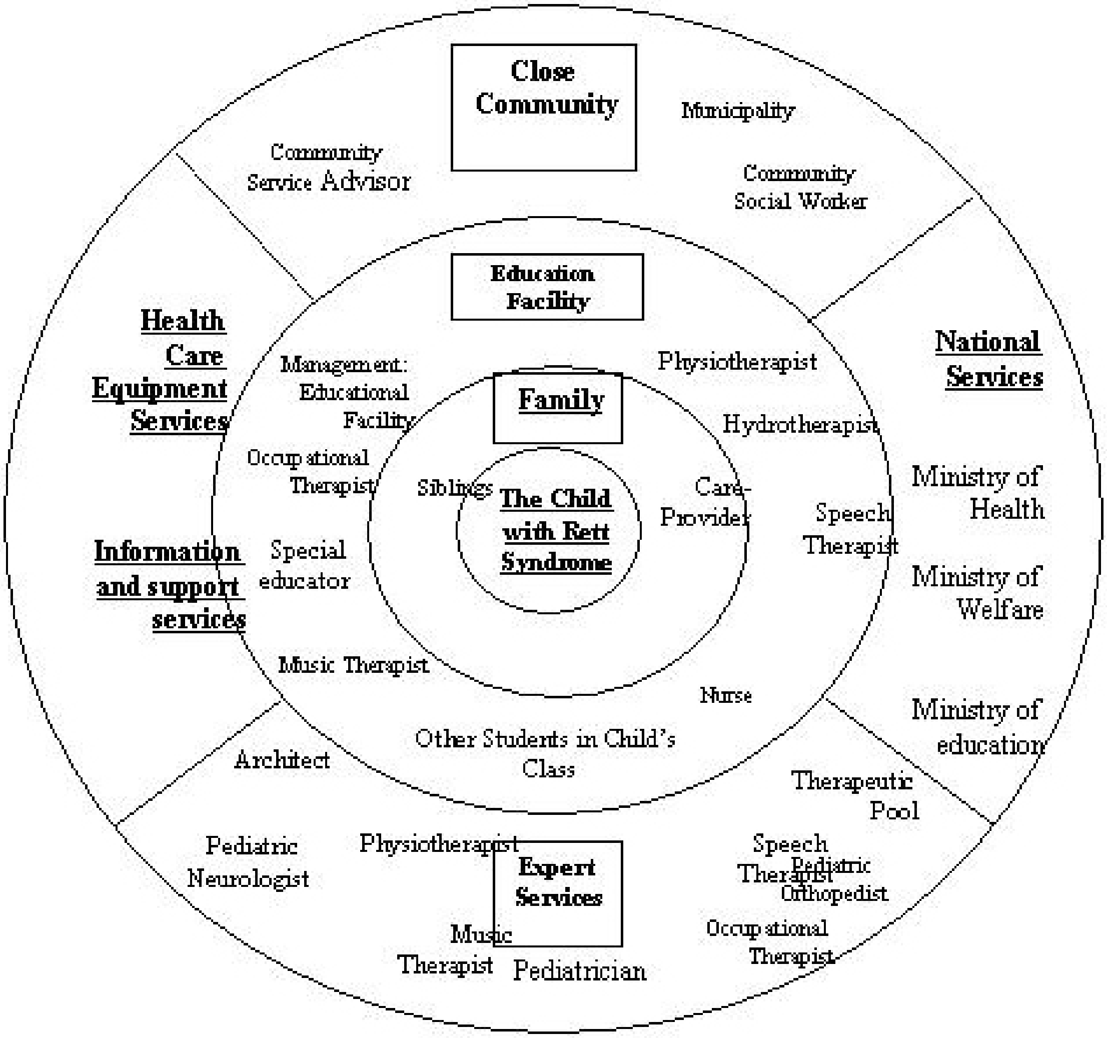

**FIGURE 1.** Schematic drawing of the different participants influencing supplementary treatment and assistive technology for individuals with RS, presenting the complexity of this issue and the many bodies involved in administering assistive technology for the individual with RS (adapted from Hedman[13])

Professional experts can help to ensure that the device fits the client and her unique needs, recommend adaptations to use the device correctly, and provide training in for the proper and full use of the device. Depending on the type of equipment required, a qualified therapist would assess the individual and make a recommendation to the organization responsible for the provision of the equipment or to the person or agency that has requested the assessment. Generally, the following applies, but the assessment process and provision may vary in different countries:

- Occupational therapists will assess for aids to daily living. These include wheelchairs, mobility aids, and specialized chairs; feeding accessories; bath, shower, and toilet aids; stair lifts; hoists; etc.

- Physiotherapists will assess for movement, strength, and balance training equipment; wheelchairs, mobility aids, walking aids, and exercise devices.
- Speech and language therapists will assess for communication, speech therapy, feeding accessories, and training aids.
- Other relevant therapists and specialists may also be involved to carry out assessments, depending on the equipment or appliance required.

The complexity of the procedure necessitates that details are clarified before purchasing and adapting any technological aids[12] (see Fig. 1). It is important to weigh the advantages of the device. If we know how to provide a client with a bed that will keep her from falling out, a lift to prevent a caregiver from suffering backaches, or a standing frame that can help the person to avoid future surgery for lengthening of the Achilles tendon, then we have obtained our objective. Daily practice and use of suitable aids will enable the client to attain improvement and prevent deterioration.

## ASSISTIVE TECHNOLOGY AND RETT SYNDROME

The Australian RS study[14] has gathered information regarding the use of assistive technology among individuals with RS. Their finding suggested that the majority of individuals with RS are using such appliances (see Fig. 2).

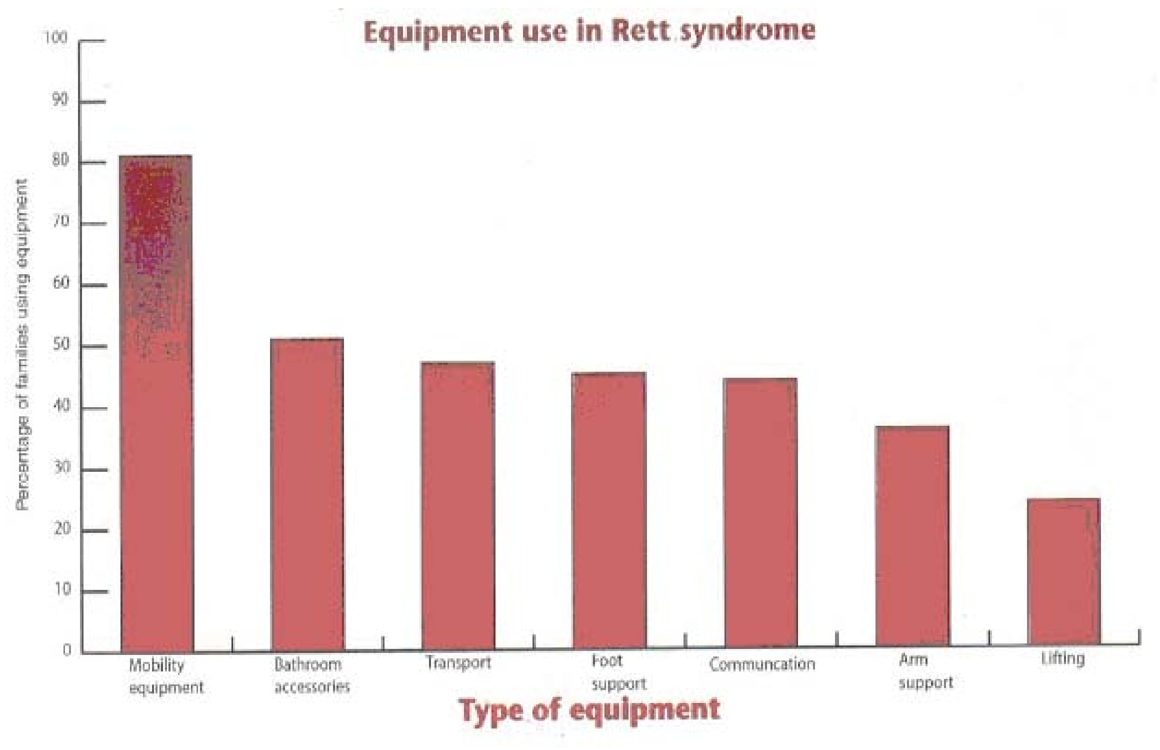

**FIGURE 2**. Equipment use in RS.

Despite the obvious need for such appliances for assisting in the daily lives of individuals with RS, many have commented that “they had difficulty in obtaining the equipment….” The commonly mentioned obstacles in achieving assistive technology were “the financial expenses and challenges associated with acquiring this equipment"[14]. Due to such difficulties, this article aims to introduce the elaborate process involved in the proper purchase, adaptation, and use of such appliances with special focus on individuals with RS.

There are ten basic steps involved in choosing or adapting assistive technology:

1. Identifying the problem
2. Determining realistic objectives in a discussion with the client human sorounding
3. Performing a functional evaluation
4. Planning the required intervention
5. Choosing suitable technological aids
6. Attaining funds
7. Delivering the equipment and instructing the staff and/or family in its use
8. Installing the equipment
9. Practicing the use of the equipment
10. Performing follow-up assessment

The stages that precede the purchase of assistive technology are critical and are therefore discussed here in detail.

## Identifying the problem

This stage is also connected to the second step of the above: defining the desired objective (the individual with RS will be able to walk independently; the individual with RS will be able to feed herself). This stage involves performing a preliminary evaluation that will enable identification of the existing strengths and difficulties, and achieving the desired objective.

## Performing a functional and environmental evaluation

This evaluation should identify challenges and ways through which they might be alleviated. This evaluation should take into account the person with RS's abilities and difficulties, her human support factors (such as caregivers, parents, siblings, and the educational staff), her various living environments (home, school, community), and the frequency and mode of transportation between these environments[12,15].

## Determining realistic objectives

After the preliminary evaluation, objectives that can be achieved without the use of aids can be determined. (The basic concept is that assistive technology should be used as seldom as possible, but according to client capability and demand). Only when the therapist has identified existing difficulties in attaining functional abilities through independent function and only after the thorough investigation into the use of adaptive techniques has been explored, then the use of assistive technology should be considered.

## Planning the necessary intervention

The evaluation helps us to decide if it is preferable to begin an intensive rehabilitation program to increase the person's independent functional abilities or to conclude that the objective cannot be attained without technical aids. It is important to assess each person and to choose treatment strategies that are compatible to her personal needs and abilities[16].

## Choosing suitable assistive technology

After the completion of the evaluation and conclusion regarding the need for assistive technology, we must search the available selection and find the aids that are suitable to the needs of each person within her various life surroundings, modes of transport, and other aspects. If professionals or institutions who deal with assistive technology have not yet been consulted, this is the time to get them involved. Choosing assistive technology demands a wise, educated decision, based on consultation with a variety of experts[17] as can be viewed in Fig. 1. Attention should be paid to the following issues:

- Compatibility — With the patient's present needs; her age and her expected future development.
- Choice — Assess the numerous choices of aids that are available and consult with a professional. Sometimes equipment can be borrowed in order to assess its suitability before purchasing it. Due to the unbalanced sensory system of the individual with RS and her difficulties adjusting to new situations[18], this option is strongly advised.
- Safety and quality — Ensure that the device is stable and has no dangerous, sharp corners or any small parts that might get lost or swallowed. Preference should be given to equipment that bears a stamp of approval by official agencies.
- Adjustments — Consider the number of times it will be necessary to readapt the equipment during the period of time it will be in use. Can this be done easily? Will these changes have any affect on its usability or safety?
- Comfort — Can the equipment be used comfortably? Can the client easily utilize it? Could the material from which it is made endanger the user? Is its structure comfortable? Can the caregiver operate and maintain it easily?
- Acceptance — It is important that the equipment be acceptable by the individual with RS who will be using it, and by her caregivers and parents. Rejection of assistive technology by users for various reasons will quickly terminate its use even if the device itself is of assistance to the user. The individual with RS's opinion could be examined through the use of Assistive Augmentative Communication (AAC).
- Transport and storage — Equipment must be of a size and weight that can meet everyday needs such as transport and/or storage. (A heavy wheelchair or a large rollator maybe abandoned due the difficulty of transporting them on a daily basis.)
- Cleaning — Ensure that there are no small cracks or areas that will be difficult to keep clean, leading to abandonment of the equipment due to inadequate appearance or smell.
- Repair and replacement parts — Check the cost of replacement parts before they are actually needed in order to avoid unexpected expenses later on.
- The vendor (equipment supplier) — It is important to verify the trustworthiness of the vendor with other clients and get answers to important questions such as: Will maintenance personnel come to the client's home or must the equipment be brought to a repair shop or laboratory for repair? Are replacement parts readily available, or should they be imported from abroad, thereby lengthening the duration of each breakdown? It is important to clarify the previous track record that the supplier has with this type of technology. Can they insure the equipment against damages? Do they offer training for the use of the device and, if so, what is the cost of such training? Is the device already in stock, or will there be a wait of several weeks or months until it arrives?
- Instruction — Is there a need for instruction before using the device? How long does the instruction take? What are the cognitive demands of instruction and who will receive it (the individual's caregivers, parents, or the user herself)?
- Attaining funds — Many assistive technology devices are extremely expensive. Funding for the equipment and the sources available should be assessed at the beginning of the process! A situation where we have completed the assessment only to find that the necessary equipment is beyond the financial means should be avoided, since it will cause disappointment to all involved.

- Delivering the equipment and instructing the caregivers and/or family — As mentioned earlier, sources of information should be clarified before purchasing a piece of equipment. In many cases, renting a piece of equipment for several weeks can help to determine if it is suitable for the client. If an aid is purchased for an individual with RS, it must be ensured that all persons involved in her care receive clear instructions from the manufacturer and the involved therapist regarding its use, operation, and maintenance. In some cases, there is no specific available device that can exactly provide the user's needs. When this is the case, it should be clarified in advance whether the manufacturer can provide special alterations for the equipment that are unavailable when buying it and how much these alterations will cost. If the manufacturer cannot make the necessary alterations, help should be sought from organizations/experts that provide special adapted equipment which can not be readily purchased.
- Installing the equipment — The device (powered wheelchair, elevator, or adapted vehicle) should be installed by an experienced service person, and a warranty should be obtained for the installation.
- Practicing the use of the equipment — Any new piece of equipment demands a period of adaptation, particularly when dealing with individuals with RS who might show difficulties adapting to new situations. Often, users are unaware of the overall potential of the device and use it inappropriately due to insufficient initial practice and training[19,20].
- Follow-up assessment — A trained professional should be invited for a follow-up examination about 2 weeks to 1 month after the assistive technology device has been put into use, even if it appears that the purchase and choice of equipment has been carried out successfully. This visit is intended to ensure that all persons involved are acquainted with the device and know how to operate it in normal circumstances or in an emergency, and that the device is providing suitable aid for the user with RS.

## SUPPLEMENTARY TREATMENT USING ASSISTIVE TECHNOLOGY

As previously mentioned, the person with RS should be constantly supported in her functional needs and the goals of paraprofessional intervention should be carried out continuously in order for the client to gain maximal therapeutic results. Such intervention is referred to as a supplementary management program and is performed by her human milieu of support during times when paraprofessional intervention is not directly applied. This form of intervention can be introduced by using different assistive technology devices and the following part of the article will screen different aids in accordance with different postures of the individual with RS. Photos will be presented mostly when the assistive technology device in question or the method of use is unique for this population.

### Lying Down

Since 15–50% of individuals with RS are able to walk[21,22], hopefully they will spend only a small amount of time lying down.

#### *Prone Lying*

Prone lying can be utilized for improving and encouraging weight bearing on the arms and strengthen trunk extensors. This position might inhibit stereotypical hand movements, as well as increase sensory information from the upper limbs, thereby enhancing body scheme. Prone lying should be encouraged before meals, or before individual intervention sessions, during which the person with RS is expected to use her hands. For a person how is a constant wheelchair user, lying on the stomach can be employed as an alternative position to cut down periods of prolonged sitting, thus avoiding the development of decubitus (pressure sores) and preventing flexion contractures of the hips. Using a wedge (see Picture 1)

can encourage safe, active play for children who suffer from extreme fear of movement and are afraid of transitioning from one position to another.

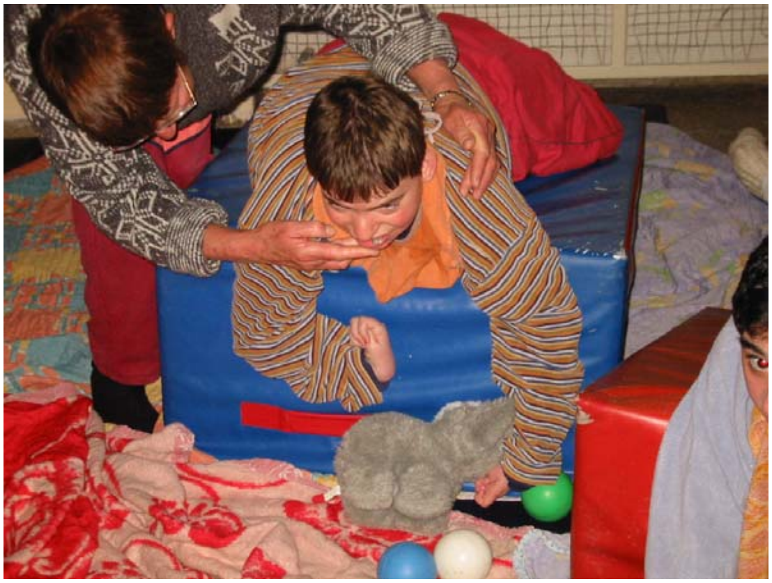

**PICTURE 1**. A child on a wedge.

Lying on the floor is a good position to encourage social contact with younger siblings; a point of strength and motivation for most individuals with RS. This position can also be utilized to encourage and motivate play. If the mat that the person is lying on is covered with interesting, colorful material, she will attempt to change the angle of her head and body and try touching it. Play can also be encouraged by placing several toys that she likes nearby, placing colored stickers on her hands, placing a bracelet with bells on her wrist, or any other stimulation that will encourage deliberate/intentional movement of the hands and fingers.

Lying on the stomach is a relatively safe position for an ataxic person who has difficulty controlling her movements. This position encourages the individual to use her hands while her body is fully supported. It will improve joint stability as well as contribute to normalizing muscle tone, while strengthening the upper back muscles. If we place a wedge under the person with RS, it will be easier for her to use her hands to push her body in to maintaining an erect position. The person with RS can be encouraged to bear weight on her arms with relative ease by placing inflatable plastic swimming buoys or elbow supports on her forearms (see Picture 2).

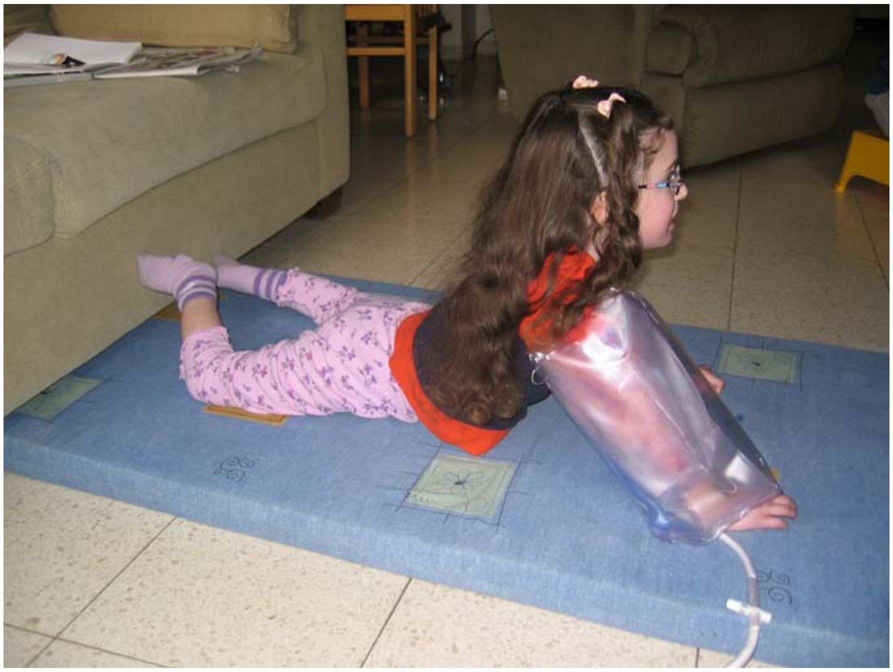

**PICTURE 2.** A child with plastic elbow supports.

It is important to place individual with kyphosis or ones that constantly use a wheelchair (over 10 h/day) in this position often. In the case of clients who have already developed flexion contractures of the hips/pelvis/lower back, the use of small pillows under the abdomen is recommended at initial trials in the new position, gradually decreasing the pillows in size. These could be placed under the pelvis until the individual with RS is able to assume a straighter position on the mat or surface with minimal discomfort (see pictures).

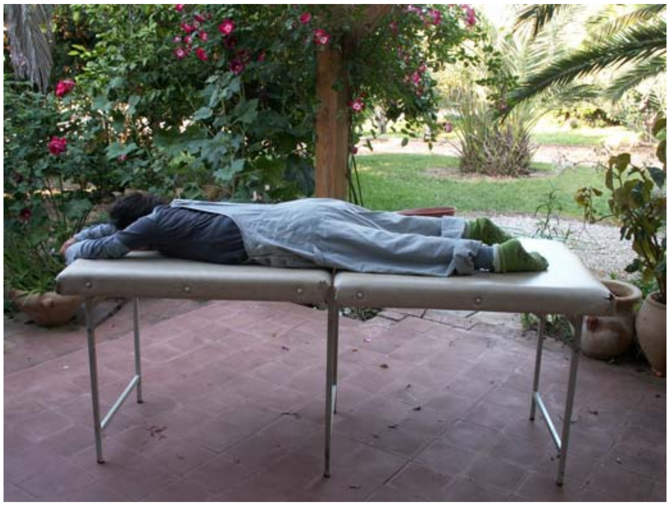

**PICTURE 3**. A child on the stomach on a therapy bed.

The very hypotonic individual with RS might show considerable difficulty in maintaining an erect position while sitting. Exercising her upper trunk muscles might be initially possible only in prone position. In this posture, with the aid of an interesting motivational factor (a toy, TV), the person will strengthen her upper back musculature. Such a program if performed on a daily basis and gradually increasing in duration might later result in improved sitting ability.

Another possible position would be to place the person with RS on a massage bed with a hole for her head, with her hand on two sides of the head. In this posture, her whole body is erect, including the shoulders and upper torso. This position is recommended due to the tendency of individuals with RS to attain a kyphotic posture due to the constant hand mannerisms (see Picture 3).

Caution: When assuming the position, it is important to prevent the neck from achieving a hyperextended position. This can be achieved if the individual's focus of interest (such as the TV screen) is properly situated. For instance, if the surface she is lying on is a bit higher than the TV's level, the person will also be using her upper trunk musculature in a straight posture, holding her head in an erect position. Another solution could be placing the TV under the bed.

### Supine Lying

This position can be used to attain numerous objectives, such as socialization. In this position, the individual with RS can see playmates, and young siblings can be encouraged to make close, intimate contact with their sister. Shiny toys that will draw visual attention or make noises, plush animals, or other objects that will encourage her to reach out can be placed above the person with RS when she is in supine lying.

Lying on the back is a good starting point for rolling the client from side to side. This type of movement could be a part of a stimulation plan intended to reduce fear of movement and accustom the individual with RS to movement (many individuals with RS are afraid of movement and this fear is expressed by an anxiety towards external manipulation)[23]. Moving the person from side to side while she is on her back causes massive tactile stimulation that helps her to construct body schemas (particularly if she is active in performing part of the movement). Such exercise might make it easier for her to cope with manipulation by an external facilitator, moving her body at other instances. The straightening of the thighs and knees and elongation of the anterior pelvis and stomach muscles are also attained in this position. Therefore, supine lying is an important position for individuals who spend an extended duration of time in a wheelchair. From this position, the person is able to change to side lying position.

### Side Lying

Lying on one side also achieves various treatment objectives. This position can be used for individuals who spend most of the day in a wheelchair. It is a good transitional position, and can be used to prevent pressure sores in the buttocks area. It is also another position from which the individual with RS can view the world, experiencing a slightly different angle. Lying on one side encourages the raising of the head to the central axis, thereby strengthening the side muscles of the neck and trunk, and improving head control (poor head control is typical of individuals with hypotonia). Maintaining this position while the caregiver/therapist/parent performs slow rhythmical movements will help in balancing muscle tone. Therefore, individuals who are extremely spastic or hypotonic can gain advantage from being placed in this position.

The static position of the lower extremities resting on the mattress enables a good, stable base for movement of the upper extremities and trunk against it. Therefore, this position can be introduced as part of a "range of motion" program for the individual with RS. Side lying stabilizes the body of the ataxic individual and might enable successful attempts of hand usage during play activities. Therefore, it is also important to place games, favorite toys, a mirror, family pictures, favorite foods, or the family pet in front of her when she is lying in this position in order to enhance functional hand movement.

Side lying is extremely important during instances when the shortening of soft tissues at the trunk is observed. If this condition is not dealt with, the client’s back muscles on one side may become shortened, gradually leading to a loss of erect postures. Such a situation might terminate in loss of independent seating, standing and\or walking, and the development of scoliosis. The person with RS should be placed in this position using special support, such as a small cylinder made out of a pillow, blanket, or rolled-up towel, with the affected side upward (i.e., an individual with a right-oriented scoliosis should be placed on her right side). This position mechanically elongates the tissues, thereby preventing or at least inhibiting the shortening of the trunk muscles.

A good, permanent side position can also be achieved at night by rolling a sheet around the client while she is lying on her side[24]. If the individual with RS does not mind, she might be placed in this position while resting or sleeping, and by implementing such a program, the time length invested in supplementary management program will be greatly extended. Another way to lengthen the treatment time for lying on one side is to use a U-shaped pillow to keep the person in the desired position. Using this type of pillow is an efficient method of treatment when curvature of the spine develops (see Picture 4).

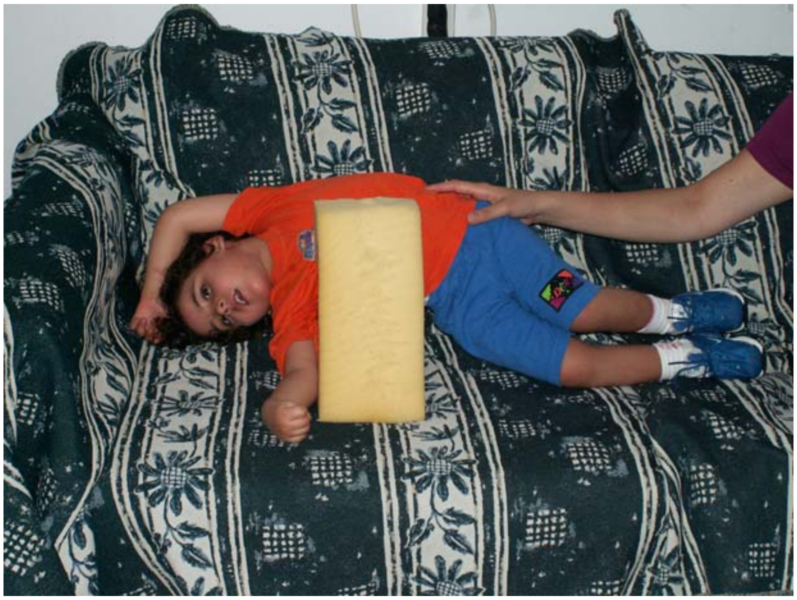

**PICTURE 4**. A child side lying with a U-shaped pillow.

This type of pillow can be made from stiff foam rubber, Styrofoam, or soft plastic. The amount of firmness depends on the user's reaction and the amount of time she spends lying on that pillow. Lying on one side is a good starting point for getting up onto a side sitting position. Side sitting usually involves weight bearing on the arms, which is another important therapeutic element for individuals with RS.

## Sitting

The objective of placing the person with RS in a sitting position is to provide external support and help her to function, despite her various limitations. Proper sitting solves existing problems, improves stability, and lessens the person’s total disability. The objectives of correct sitting are[25]:

- To improve manual function
- To improve lung function
- To improve motor control of the mouth, thus improving eating and drinking
- To normalize muscle tone and improve balance of the trunk
- To improve proper movement, thus improving function
- To allow more time for leisure and socialization
- To improve the trunk and head control
- To improve quality and quantity of vocalization
- To enhance comfort and improve quality of life

A "seating system" is a general term describing any chair in which the person sits. Before adapting any type of chair, it is important to consider and analyze the user's everyday physical needs, as well as those of her caregivers and members of her family. Different sitting options are described in the next section for possible implementation (see the guidelines for integrating assistive technology described at the beginning of this article).

## Puff Chairs/Bean Bags

Puff chairs are large bags fashioned out of cloth or leather, and filled with tiny Styrofoam balls. They can be molded into many shapes to support the person's entire body. When seated in a puff chair, the individual with RS feels secure, comfortable, and well contained. It is preferable to use this type of seating arrangement for short periods of time or for short dozing periods during the day.

It is important to remember that this type of chair decreases the individual's mobility (which is already restricted due her physical limitations). When the individual with RS is placed in a partially prone position, her trunk is bent forward into a kyphotic position, which is prevalent among this population and therefore must be discouraged.

Individuals who are able to ambulate independently should not be encouraged to use puff chairs on a permanent basis. When the person with RS is seated on such a bag, it is extremely difficult for her to get up. Since the ability to perform transitional changes is one of the first mobility elements lost by individuals with RS[26,27], such passive placement is not highly recommended for prolonged periods of time.

## Side Seater

A side seat is a therapeutic device developed by the author for individuals with RS showing asymmetry of the trunk. Such intervention should be implemented as soon as an asymmetry of the trunk is noticeable[28].

The device is designed to encourage asymmetric work of the trunk and is, therefore, an efficient tool for preventing or at least reducing curvature of the spine among individuals with RS[28]. The position of the individual with RS in this device is stable, thus encouraging hand function and play. The straps that hold the person in place do not allow mobility, therefore the seater is recommended for ambulant individuals only for short periods during the day. The height of the side support in this device should be adjusted against the height of the potential collapse point of the person's spine and should be designed to encourage active side flexion and extension (see Picture 5).

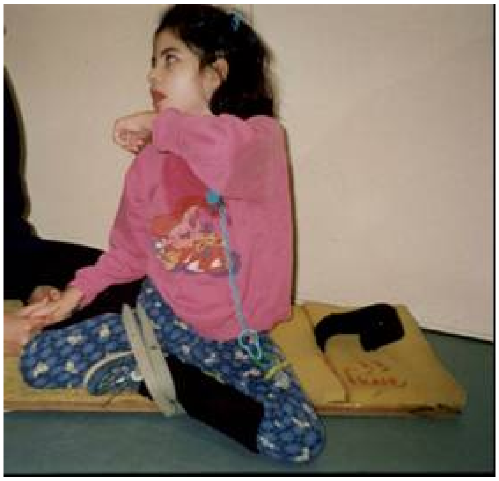

**PICTURE 5**. A side seater.

## Corner Chair

This type of chair is usually made of wood. The corner chair offers strong support for the back, and keeps it straight and perpendicular to the legs. This type of support to the trunk frees the hands to perform manual activities. A table (with a rim around it to prevent toys\communication devices, etc. from falling off) can be attached to the chair in order to encourage communication and play. The height of the back can be changed according to the height of the seater; therefore, this type of chair is also suitable for individuals with poor head control. The chair holds the person with RS in a full, seated position and thereby stretches the muscles of the lower back as well as the hamstrings. The chair keeps the person sitting in a stationary position and offers security, but on the other hand, prevents movement. This type of chair may assist in giving the user with RS a symmetrical proprioceptive stimulus, establishing midline perception. Therefore, it could be used as a preventive measure for the development of skewed midline, leading to the development of scoliosis as suggested by Hanks[27].

The downside of this chair is that the user is unable to get herself out of the chair. It is not recommended to use this type of chair for long periods of time for a person with self-ambulation abilities. When using this chair, the user must be strapped in so that her pelvis is at the back of the chair and her knees are only slightly bent. The caregiver/therapist/parent should be advised that if a waist belt is not used to hold the seater in a proper seating position, she will slip forward, thereby encouraging the progression of a kyphotic curvature of the spine.

## Knee Chair

This type of chair (see Picture 6) is built so that it creates lumbar lordosis by tilting the pelvis forward (producing an anterior pelvic tilt). It offers support for the knees and pelvis, but leaves the back free for activity while being supported due to the pelvic posture. Using such a chair should be considered as a means for preventing curvature of the spine and kyphosis[24]. These chairs can be used with rocker bottoms, intertwining stability and security with motion, and can be controlled by the user (the amount of movements caused by the users trunk sway will influence her rocking). This type of chair could be considered for use as a possible intervention management device for the person showing fear of movement (initially placing it over a thick carpet, gradually moving the chair to a firmer floor).

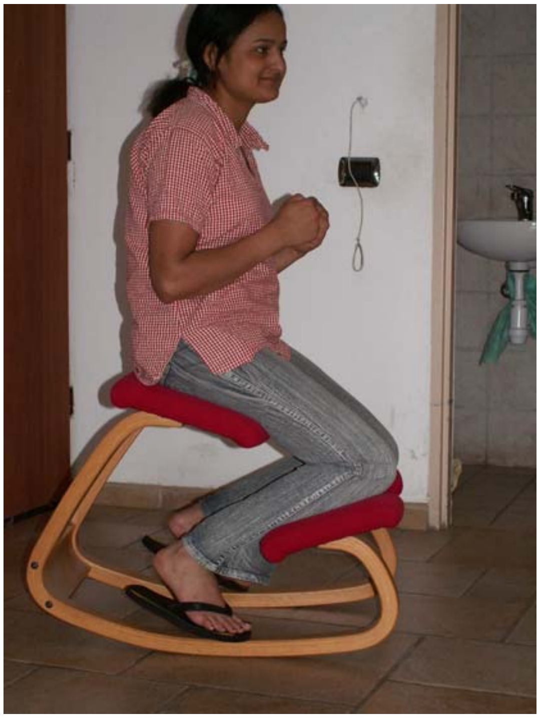

**PICTURE 6**. A knee chair.

### *Trip Trap Chair*

This type of chair is recommended by many parents. It is aesthetic, it enables movement while the person is seated, and its dimensions can be changed easily as the child grows, or in order to adjust it to the height of a family table (see Picture 7).

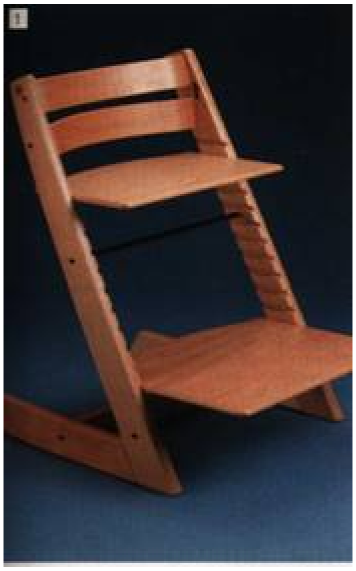

**PICTURE 7**. A trip trap chair.

This type of chair is suitable only for those with full trunk control, since no trunk support is present. The disadvantage of this chair is that its footrest is usually higher than the floor, creating a sort of a step, which most individuals with RS find difficult to descend from. This step impairs the user's independence and movement. In the case of mobile individuals, it is preferable to consider a chair without a footrest so that the user is free to get up independently and walk away from the chair. Another possibility is to add a small staircase to this type of chair, thereby producing an opportunity to practice stair climbing and descending before and after every meal.

### Stools

Individuals with RS have a tendency to lean against the nearest stable object around them. This inclination also includes the backs of chairs, when seated. The author's experience has shown that in some individuals with RS, this phenomenon is exaggerated and leads to a permanent tendency to lean back at all situations, resulting in extension of the trunk when standing and walking, up to a point where independent walking and even seating is lost. The ability to make transitions and change positions is one of the first motor functions lost by the individual with RS[26,27] and strong measures should be taken to keep the client as active as possible[2]. Seating the individual with RS on a stool is helpful in achieving such goals. Sitting on a stool obligates the individual to actively preserve balance of the trunk and prevents her from reverting to a permanent reclining position.

If such level of trunk control can be achieved by individuals showing severe scoliosis, an uneven active seating surface can be introduced as a corrective measure against the development of scoliosis (see Picture 8).

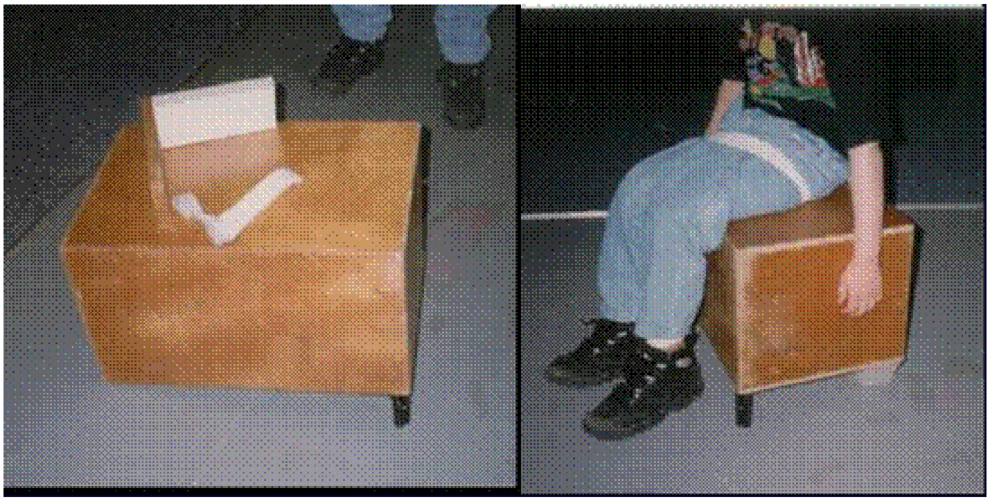

**PICTURE 8**. A child sitting on an active uneven stool.

If the individual with RS possesses higher degrees of stability, it might be possible to consider the use of a T chair as a regular practice.

### Regular Chairs

Any chair must be adapted to the specific needs of each user. For this reason and due to the fact that each individual with RS is different, there is no standard chair for them as a group. The basic guidelines when considering a chair for your child/client should be:

- Individuals with RS tend to lean towards available support. Therefore, any support, including chest vests, should be avoided as much as possible and active seating, even for parts of the day, should be constantly encouraged and implemented.
- A belt should always be added as a means for appropriate pelvis positioning and if the seater with RS is prone to epileptic seizures, a heavy-duty knuckle should be added to the belt as a safety measure.
- The client's feet must touch the floor in order to avoid shortening of the Achilles tendon, and to provide constant sensory input and supply stability for function.
- The height of the footrests should allow independent climbing and descending for individuals able of such performance.
- Side supports might be added, if necessary, according to the person's trunk control and spinal deformities (although even in severe cases of scoliosis, it is highly recommended to try to achieve unsupported erect seating as a daily intervention program).
- The table at which the individual with RS is seated should not be too high as to limit hand function, or too low as to encourage bending forward of the trunk, which can promote a kyphotic posture of the upper back. The rule of thumb is that the working surface (table) should be 5 cm (2 in.) lower than the elbows when the arms are held against the user's body. This height will enable her to control objects that are on the table and to engage in activities such as finger feeding, reaching a cup (suited with a straw), and operating a communication board or a computer switch.

A heavy, stable chair should be considered for heavy individuals who are prone to epileptic seizures or are usually engaged in rocking and swaying movements. In order to add stability in such cases (epilepsy) without considerable addition to the chair's own weight, additional horizontal and vertical supports can be added to the base of the chair to keep it from toppling over.

When a person with RS displays the first signs of curvature of the spine, intervention should be suggested immediately[28]; asymmetrical corrections can be made to the side of the chair by raising the affected side (if the individual is showing right scoliosis, then the right side of the chair should be elevated to tilt the individuals weight to the left) and adding a side support at the level of the tip of her scoliosis (see Picture 9-10).

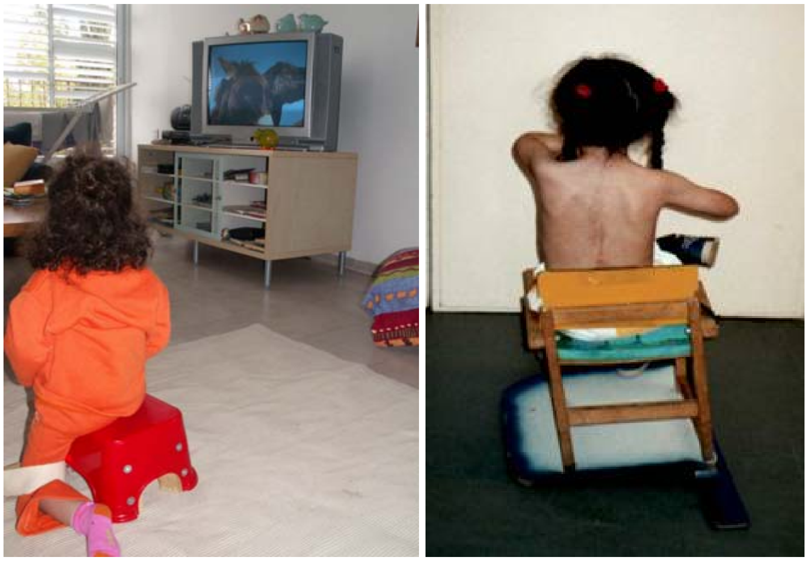

**PICTURE 9-10**. children with RS sitting on specially adapted chairs.

The point of this exercise is to encourage asymmetric muscle work against the direction of the curvature[29]. If the cause of the scoliosis is sensorial in origin, other measures should also be taken[30,31]. Usually, we would hope for the individual with RS to be as active as possible at all times, or at least involved at a part-time activity program (such as an active seating arrangement). At the same time, we want her to keep her concentration focused on educational intervention and enjoy her meals as well as her recreation time (mostly spent in a comfortable chair). Furthermore, we do not want her to miss out on outdoor activities involving family and friends that might require walking beyond her capabilities (in such cases a wheelchair can be implemented). Therefore, in specific situations, the person with RS may need an array of seating arrangements to meet her different daily requirements (see Pictures 11–13).

### Wheelchairs

The initial decision to use a wheelchair is an extremely difficult one at most times, and must be assessed after consulting with the parents as well as with the management staff. It is often easier to accept the first wheelchair if it is similar to a child's stroller, is non-disability oriented, and appears less threatening. A wheelchair, when appropriately administered, provides a good sitting position and mobility for the user who presents with limited ability to walk. It also provides support for individuals with postural problems or deformity while improving comfort, independence, and safety during seated activities[32].

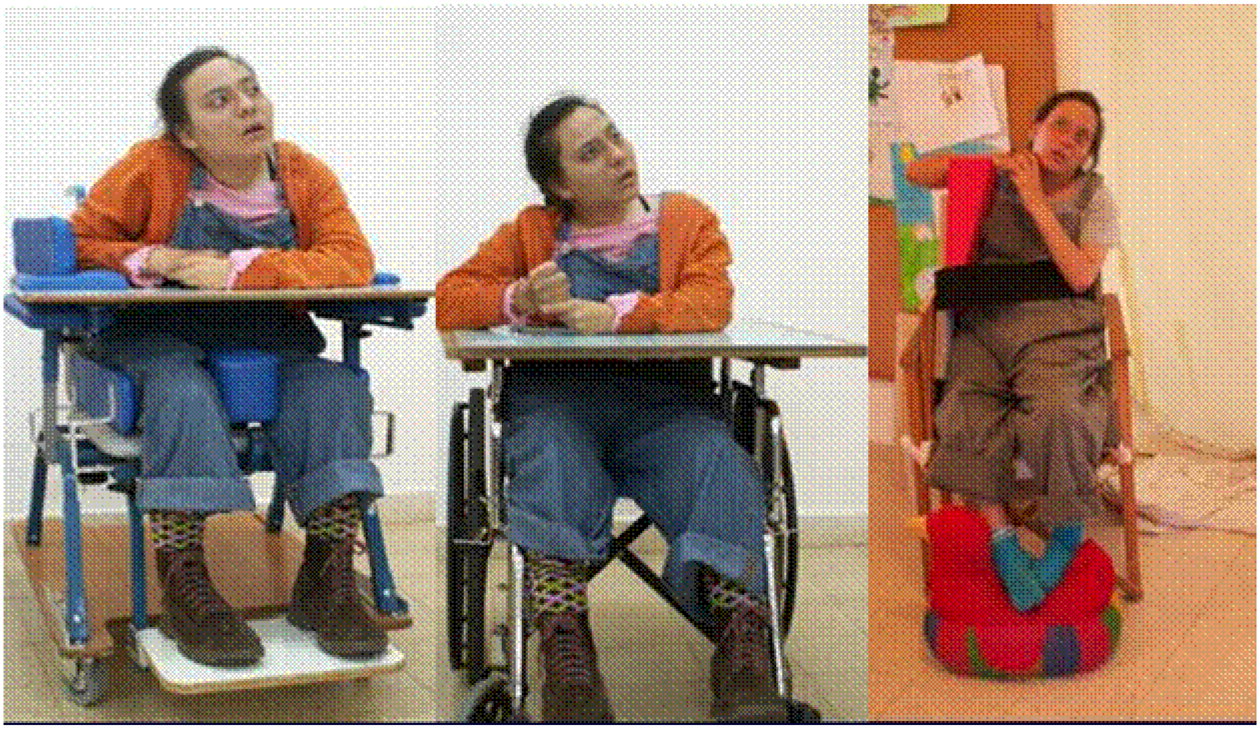

**PICTURE 11–13**. A child with three different seating arrangements.

The author would like to emphasize here that moving from a stroller to a wheelchair should not mean giving up on walking altogether. One of the children with RS whom I personally cared for received her first wheelchair at the age of 8 years due to her large physical stature and the her body extreme body sway making it difficult to control her movement through assisted ambulation. She is now 18 years old and still performs most of her daily ambulation by means of (assisted) walking.

Guidance from a therapist knowledgeable in wheelchairs and seating systems is advised whenever a client is prescribed a wheelchair, but it is particularly important for individuals who are at risk of pressure sores, show postural problems and/or difficulty with sitting balance, and will use the wheelchair most or all of the time[1], as is the case with many individuals with RS. As mentioned before, each individual with RS is different from others with the same condition and, on top of that, the symptoms and phenotypic expression of the syndrome might change with age. Younger girls are mostly hypotonic and walking may be difficult for them; 15–50% of the girls with RS do not develop ambulation, and are therefore in need of a proper seating arrangement appropriate for long seating durations and transportation[22]. At an older age, some of them develop high muscle tone while others will show fluctuating/distonic muscle tone[33]. The first seating arrangement is usually a suitable baby stroller. Some young individuals with RS who are able to walk may also need a stroller for outdoor activities with their family; 25–85% of individuals with RS are expected to lose their ability to walk at a later age (when they reach stage IV of RS), therefore possibly needing a wheelchair[22,33]. The type of chair and any personal adaptations are determined by the user's physical condition and needs. There are three main types of manual wheelchairs:

1. The basic wheelchair includes a folding sling seat and has removable footrests and armrests, yet sling seats may create discomfort, in the long run, and can make it difficult to change position, increasing the risk of pressure sores[1]. Due to the slim stature of most individuals with RS and the potentially long durations of seating, a linear insert is recommended over the basic chair. Such an insert presents a stiff backrest and seat, supplying stability and support. When a linear insert is not suggested, a seat cushion should be used with the wheelchair. Special air and gel cushions are available to reduce risk of pressure sores in patients with limited ability to shift their weight while seated[32]. This type of chair is suitable for individuals with minimal deformities and for those who present good trunk control.
2. Wheelchairs with an adapted setup id usually added with a modular insert. Other supports, such as a headrest, arm supports, or a pommel (knee separator), can be added. This type of chair provides external support and stability, which (in some cases according to individual needs) may be asymmetrical, in order to adapt to the user's body curvatures. In this manner, pressure is distributed throughout the body, reducing the risk for developing pressure sores. This type of chair is padded with various combinations of foam.
3. Custom-molded wheelchairs provide a seating arrangement totally adapted to the curvatures of the seater's body and offer the most stable form of support. When this type of seating is well adapted, it enables the greatest distribution of pressure support and containment for the body. A great deal of time, knowledge, and experience is required to produce and manufacture this type of chair. Custom-molded wheelchairs are made by using various methods of construction such as vacuum pressure or through the use of a mixture of chemical materials. They are seldom prepared on site, and usually a mold of the user's body is sent to the factory for the assembly of such a seating arrangement.

In all types of chairs, proper positioning is important, but with this type of chair (custom molded), it is crucial that the person is always seated correctly since any change of position is liable to cause discomfort or pressure sores due to lack of compatibility between the chair and the user's body position. Due to the inability of most individuals with RS to convey their distress verbally, this type of seating arrangement should be accompanied by intense staff guidance. Evaluation should be performed by a physiotherapist using his/her knowledge of anatomy, kinesiology, biomechanics, and principles of neuromuscular control and of stability and function[12,15,25].

The author is confident in the proficient skills of physical/occupational therapists who are involved in the assembly of the appropriate seating system. Therefore, only specific elements regarding special issues typical of the client with RS that might effect the construction of such a seating arrangement will be mentioned in the section below.

- The final decision regarding the purchase and adaptability of the seating system for the individual with RS and her daily activities should be made after a comprehensive examination as well as consultation with parents, caregivers, teachers, and managerial staff.
- If severe, uncontrolled seizures are present, proper measures should be taken (such as a chest vest or an upper trunk belt) to ensure the user's safety.
- A table is of extreme importance in order to allow the user with RS to bear weight on her hands, for placing a communication board, for play-related activities, and during meal times. Due to involuntary hand movements of the individual with RS, it is recommended that the table should have a high rim around its edge to prevent items from being accidentally pushed over.
- Some individuals with RS start as hypotonic; gradually some of them (about 40%) will develop spasticity and/or rigidity mostly initiating from the shin muscles. About 30% will remain hypotonic, while the rest will show fluctuating/distonic muscle tone[33]. Due to such changes over different age periods, the seating system should be constructed while taking the client's basic muscle tone into consideration and should be adapted according to the changes observed in muscle tone (regarding the amount of support, type of wheels, etc.).
- Due to the above mentioned changes in muscle tone[33], to sensory abnormalities[34], and to functional fluctuations typical of the individual with RS, the aptness of the seating system should be assessed at delivery and reassessed over a period of 6 months of use. This additional checkpoint is intended to ensure that the supplied system is fully innate with the flexible nature of RS.
- When the client is showing a tendency towards passivity and with the assumption she is intended to stay seated for long durations during each day, the chair should be fully adapted to pressure sore prevention (regarding appropriate cushioning and the ability for tilt-in-space and recline of the seating system). Such adaptation is necessary due to sedentary behavior and to slim body composition typical of 85–90% of all individuals with RS[14]. The fact that this situation deteriorates with age[34,35,36] augments the management of individuals with RS as a population at risk for developing decubitus.
- When the individual with RS is to use her seating system for many hours a day, the caregiver should be responsible for changing weight-bearing areas, and alternate between different functional seating and resting positions according to a preset program designed by the physical or occupational therapist.
- It has been occasionally reported that tilting the chair forward about 5 degrees improves head control, decreases deviation from the center line of the body, and increases function[24] therefore this option should be considered for the individual with RS.
- Some individuals with RS, mainly those who lack mobility and are extremely hypotonic, have a tendency towards external hip rotation[37]. This sometimes causes hip anterior displacement and is liable to result in instability of the hips, which may become painful and prevent ambulation. When such a condition is present or is expected hip side supports should be used.

## Motorized Wheelchairs

Given the amount of involuntary movement presented by individuals with RS, as well as the difficulty in hand use, one might think a powered chair is out of the question for this population, yet problems with upper extremity coordination could be accommodated through specialized controls[1]. Using head controls is one possible solution for granting an individual with RS the ability to propel a powered wheelchair independently. There are two reports regarding the use of motorized mobility by individuals with RS. One of them used a set of head controls the other four jellybean switches[38]:

> *Joanne and her family started thinking about getting a powered wheelchair, when she was around four years of age as suggested by her physical therapist. Learning how to use*

*the motorized wheel chair was done through "simulation training" under the supervision of a physical therapist for several months. After a period of time and a lot of convincing, Joanne got her own power chair, with three jellybean switches for driving, and an over-ride switch for the adult. Joanne has had her powered chair for two full years and she loves having her power chair even if it is something she does not use every day – it is something that is fun for her and that can give her a feeling of independence, yet it requires her attention and focus. Joanne is never left completely alone, but driving a powered wheelchair gives her the chance to make some choices without having to pass them through someone else. Her friends are envious of Joanne, because she can drive[38]* (see Picture 14).

In light of such a report, the possibility of an individual with RS using a motorized wheelchair should not be ruled out beforehand. Motorized wheelchairs and scooters are becoming increasingly common. Such devices are most helpful for individuals challenged by community mobility rather than in-home mobility, and present sufficient control to enable independent, safe usage. In addition, if availability of a motorized wheelchair/scooter may increase participation of the individual with RS in activities that would be otherwise unavailable, its use should be considered. Secondary possible outcomes such as an increase in overall activity levels and a decrease in depression has been observed in other populations due to the use of powered mobility and should also be taken into account[31].

A wheelchair with sitting-to-standing position change enables the integration of two assistive technology devices into one and can be relevant when storage room is limited and the needs of the individual with RS necessitate both a transportation option as well as an enrolment in a daily standing program. Both manual and motorized wheelchairs are presently available with the ability to change the position of the user from a seated position to standing. The utility of the sitting-to-standing option for the average wheelchair user is unknown and these instruments cost considerably more than a standard wheelchair[1].

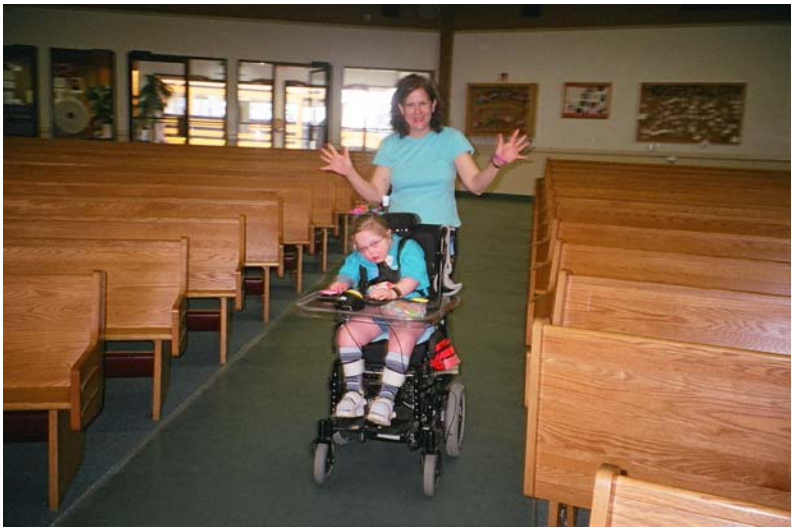

**PICTURE 14.** A child with RS driving a motorized wheelchair.

## Standing

Standing is an important starting position for daily life and function. Some individuals with RS may present difficulties in standing and walking, or cannot perform these functions at all. This constitutes a primary debilitating condition on the functional as well as the social level of these individuals, necessitating therapeutic intervention.
Standing presents numerous advantages[39]:

- Standing encourages weight bearing, thus reducing the serious effects of osteoporosis[40,41]. Individuals with RS are known to have low bone mineral density from a young age[42,43], therefore it is especially important to suggest a daily standing program for non-ambulant individuals as a preventive measure.
- A regularly executed standing program may help to prevent contractures at the knees and hips for a wheelchair-bound individual. An individual with RS that uses a wheelchair for long durations daily should be walked on regular basis[24] or, if not applicable, should at least be incorporated in a daily standing program.
- Standing is a muscle-tone-normalizing action[39]. Since individuals with RS present a diversity of tone changes[33], a person incorporated in a standing program will have the advantage of reduced tonal fluctuations.
- Standing improves the flow of blood to internal organs[39], thereby improving their functions[44,45]. Individuals with RS suffer from an array of intestinal problems[46], and those could favorably be effected when a constant, continuous standing program is implemented.
- Standing involves a transition[47] (as opposed to sitting or lying down). For an individual who spends long daily durations in a seated position, standing is highly recommended as a preventive measure against pressure sores[48].
- Standing encourages equilibrium reactions and stability, thereby enhancing balance. Such improvements might constitute the first step towards walking for individuals with RS who are only capable of sitting and is, therefore, highly encouraged.
- Standing creates pressure directed towards the hip joints[49], thereby changing the angle of the femur from coxa valga to coxa vara, as well as building the acetabulum and preserving its healthy develpment[41]. Since asymmetry of the pelvis and hip displacement are orthopedic pathologies found among individuals with RS, particularly those who are not mobile[37], a habitual standing program is recommended for this population.
- Standing enables a change in the perspective, thus enabling the client to experience her human environment at eye level. Such a change might bear positive results regarding self-image, self-esteem, and social skills[50].
- Individuals confined to wheelchairs belong to a risk group for diminished lung function. Since standing improves respiratory function[51], wheelchair-bound individuals with RS should be introduced to a routine standing program.
- Many individuals with RS display a shortening of the Achilles' tendon due to increased spastivity. Assuming a daily standing program while using a wedge under the client's feet (see Picture 15) might prevent shortening of the triceps-sura muscles, as well as reduce the need for daily unpleasant stretching exercises. If administered intensively and consistently, such an intervention might serve the individual with RS by avoiding the need for a Botox injection or surgery for Achilles tendon lengthening.
- Numerous advantages of standing were reported for individuals with spinal cord injury[39], such as improved well-being, circulation, skin integrity, reflex activity, bowel and bladder function, digestion, sleep, and reduced pain and fatigue. Other benefits reported for this population are in metabolic functions and psychological aspects[52]. It is possible that a standing program will yield similar benefits for individuals with RS.

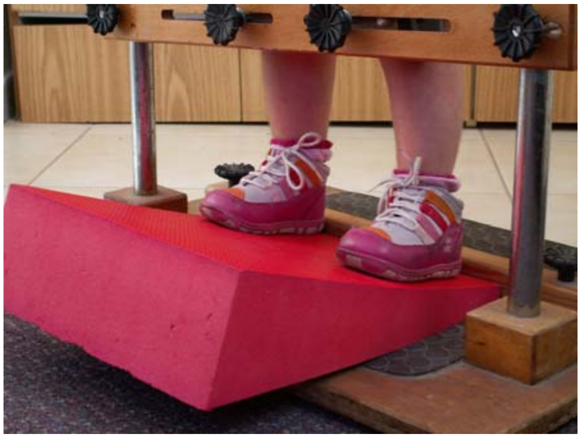

**PICTURE 15.** A child standing on a wedge.

Most orthopedic surgeons recommend that non-ambulant individuals will accumulate a daily standing duration of at least 2 h in order for such exercise to bare results. Nevertheless, a recent study indicated that even shorter periods of standing, averaging at 5 h weekly, are useful in achieving significant improvement in bone density[41].

An appropriate standing frame should be selected in accordance with the general criteria mentioned at the opening part of this article for choosing assistive technology. The basic rule of thumb is to choose a standing frame that will offer the individual with RS the minimum amount of support possible and will obligate her to be as active as possible within the framework of her limitations. Using a table while the individual with RS is standing, for toys or a communication device, is recommended as long as the user does not lean on the table, preventing her from actively using her trunk muscles.

Caution: When instigating a standing program, one should acclimate the individual with RS with the new unfamiliar situation (taking into account the difficulties individuals with RS present in such cases), as well as apply the program for minimal durations, gradually enhancing the effort demanded from the client.

## *Standing Frames*

### Tilt Board

This kind of standing frame is designed for individuals who are very heavy or hypotonic, and for those who show extremely poor trunk and head control. The tilt board is an electric or mechanical bed that can be tilted from a horizontal position to a vertical one. The client using this apparatus is placed on the board in a laying position. She is then elevated into a standing position, after being strapped by her knees and chest (and possibly with an extra safety belt at the pelvis level). A table can also be added so that the user can operate a switch or play while standing. This type of standing frame is usually used when the individual with RS is in need of massive support.

### Supine/Prone Board

This type of standing frame can be leaned towards a table, wall, or sofa with the individual with RS lying on her stomach. The amount of support this type of standing frame offers can be changed according the client’s head and trunk control, and the angle of tilt. Such adaptations allow the person using the appliance to attain an optimal position. This type of standing frame also enables the individual with RS to be socially active; it can be leaned against the dining table, enabling her to eat and communicate with other members of the family during family gatherings. Some supine boards are equipped with wheels and can be moved from place to place. The prone board is preferable over a supine board for encouraging the activation of trunk extensors, thus of great use for the kyphotic user.

## Oswestry and Adjustable Oswestry

These are two types of rigid, stable, standing frames. The simpler form is extremely rigid, made of wood, and supports the knees and pelvis. Additions can be made in order to allow chest support. The height of the table can be changed in accordance with the user's needs. The second type of standing frame (adjustable oswestry) is an improvement to the basic model and is made of stainless steel. This type of standing frame is foldable and is, therefore, easier to store. These two frames offer strong front and back support (however, they provide less support from the sides), while still enabling safe manual function. The table should be equipped with a high rim to prevent toys or other accessories from falling as a result of the client's unintentional hand movements. There are many kinds of standing frames (Richter, Bayreuth), including electric or mechanical frames that can raise the user from a seated position in her wheelchair to a standing position. All these standing frames' height can be adjusted according to the user's own height, with a range of 110–170 cm (44–68 in.). These standing frames are extremely heavy and difficult to move (even the ones that have wheels), and occupy a lot of space.

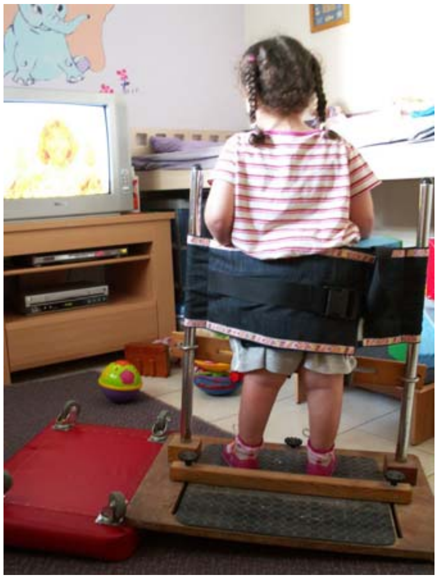

**PICTURE 16**. A child with an uneven standing frame.

## Pelvis-Height Standing Frames/Active Standing Frames

These standing frames support the user's knees and pelvis, with the back usually remaining free and active; this is despite the fact that an additional supportive belt at chest level or a table can be added. The simplest forms of active standing frames are mechanical, and consist of a board and two vertical stainless steel rods with pelvis straps that can be adjusted to the individual's height. Knee supports can also be adjusted to the appropriate height and width of each user.

More sophisticated models, such as the "Flexistand Major", are equipped with electronic lifts for lifting the user from the chair to a standing position. This type of frame can also be adjusted electronically to provide front, back, or side support, as well as adjusting the height. If the person with RS begins to develop curvature of the spine, an asymmetric standing frame should be used in order to encourage the use of muscles to counter the direction of the curvature (see Picture 16). This method has been found useful in reducing scoliotic curvature[29].

## Shin-Standing Frame

This type of standing frame was initiated by the author (see Picture 17). It is suitable for a user who basically has moderate trunk control, but requires minimal assistance or supervision in maintaining an erect posture. This type of standing frame can be made from an old pair of AFO (Ankle Feet Orthosis) splints attached to a wooden board. This standing frame supports the legs (the height of the splints, above or below the knees, is adjusted in accordance to the user's ability) and is designed to serve as a device for practicing standing and improving static balance abilities. It is difficult to control, but offers good support for the hypotonic individual who possesses enough trunk control for maintaining this posture. After the person with RS has become accustomed to this type of standing frame, adjustments can be made, such as reducing the base of support (by narrowing the space between the splints or placing them in a variety of tandem positions on the wooden base); such changes enhance the users activity level.

The shin-standing frame can then be adapted to achieve higher levels of dynamic balance by inserting a screw underneath the center of board. This causes the base to move in different directions, and standing on it becomes an active, challenging exercise. Slow, controlled movements in any direction are made possible by adding foam on the bottom of the base. The user can then feel relatively secure while moving slowly and gently, exercising and strengthening her trunk muscles and learning how to actively keep her balance, all at the same time.

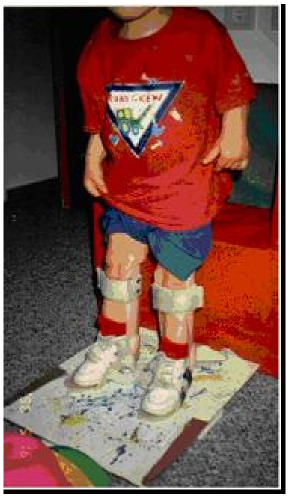

**PICTURE 17.** A child with a shin-standing frame.

## Stationary Standing Area

A standing area is a device developed by the author. It is a safe and enclosed padded area that provides the user with RS with the opportunity to stand independently and even perform a few steps while safely and independently staying erect. Such a device can be made with a large barrel (individually adapted to the dimensions of each user and her standing abilities). It allows the user to remain in a standing position without requiring constant supervision. A motivational factor, such as a TV, game, audio recorder, or communication device, can be placed within reach; so that the person standing in the device can stand or move while involved in a recreational activity (see Picture 18).

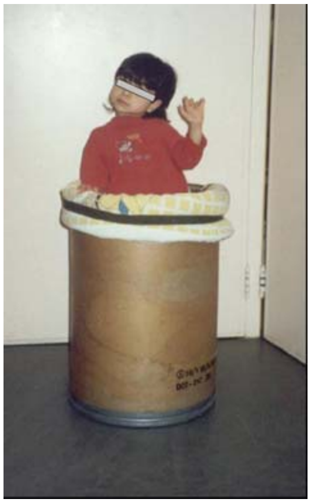

**PICTURE 18**. A child in a standing space.

## A Manual Standing Frame

This device was developed by the author especially for individuals with RS. It is made out of two poles, which the individual with RS holds with her hands. This type of standing frame is suitable for a user that has full control over her legs, but is in need of assistance for standing or walking. The person with RS can hold the standing frame by herself or be strapped to the bars as a safety measure (in cases where hand usage is not fully reliable) (see Picture 19). Side-to-side balance reactions can be achieved if the person with RS holds on to only one bar (see Picture 20).

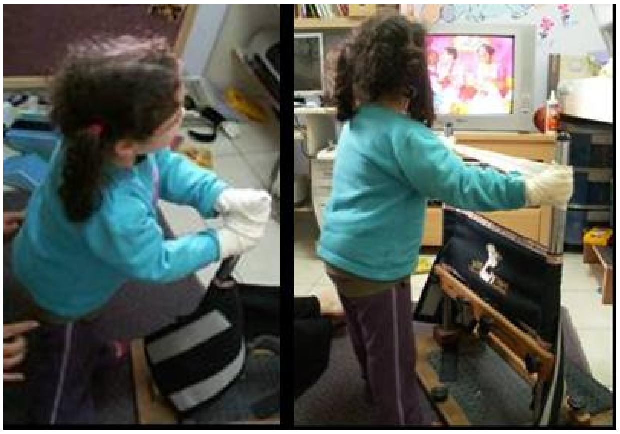

**PICTURES 19 and 20**. Manual standing frame with hands attached.

## Walking

One of the most immediate questions asked by parents of children with disabilities in general, as well as by parents of individuals with RS, is: “will she be able to walk?” Such a question stems from that fact that walking symbolizes independence and allows the individual to be embraced by society as a person with a problem as opposed to a non-mobile individual who is perceived as a person with a severe handicap[25]. Walking is crucial for any individual with developmental disability, but is extremely important for the person with RS, as well as highly recommended by experts in the field of RS[2]. If walking as an independent ability cannot be achieved, then short-distance therapeutic walking should be constantly incorporated into the supplementary management program. Walking, even therapeutic walking, has many benefits. Walking offers all the benefits of standing (see the previous section on standing), as well as the following advantages:

- Improving function and physical fitness — A daily, treadmill walking program was implemented for four children with RS for the duration of 2 months. Sessions lengthened for half an hour each, and provided the participants with comfortable walking speeds. The program was found to improve physical fitness and functional abilities of all participants[53]. Training on a treadmill has been found to encourage independent walking in other populations[54,55].
- Preserving joint range of motion (ROM) — Walking improves ROM of the back, pelvis, hips, knees, and ankles, and exercises many different joints[56]. Mobility is essential for individuals with RS who are in danger of joint contractures and should therefore be set as a primary therapeutic objective.
- Strengthening massive muscles of the lower limbs — The strengthening of muscular mass[57] reduces the secondary dangers of immobility, such as muscular atrophy. Bauer claims that the assembly of accumulating factors reducing the individual's level of activity, such as sedentary lifestyle, reduced functional abilities and physical fitness, medication, as well as other factors, presents an eminent danger for individuals with disabilities[58].
- Enhancing pulmonary activity — Using the muscles of the lower extremities, which are the largest muscles in the body, forces the lungs to work at higher capacities, resulting in improvement in lung capacity and function[57,59], thereby enhancing a general, more apt condition for the individual with RS.
- Improving heart function — Walking as an exercise of the large muscles of the lower extremities forces the heart to circulate blood to the extremities at higher rates, thereby enhancing aerobic capacity[60] and improving heart function[61,62]. It should be taken in to account that 50–100% of individuals with RS at stages III and IV present slight cardiac irregularities[63,64], such as elongated QT intervals. Therefore, any controlled physical activities that stimulate and strengthen the heart muscle are desirable, after consulting with a physician.
- Positive prognosis for scoliosis — Scoliosis has been identified in 80–85% of individuals with RS[22,65]. A negative connection was described between the person’s ambulation abilities (especially her abilities to ascend or descend stairs) and the severity of scoliosis[22,65]. Therefore, intervention aimed at improving and maintaining the person with RS’s ambulation abilities will have a positive effect on the severity of her scoliosis.
- Organization of body schemas — The action of ambulating enhances sensory stimulation of limbs and torso positioning. These proprioceptive messages are transferred to the neural system mostly when the individual is active. Therefore, walking is a continuous exercise that constantly reorganizes the neural system of the walker through different mechanisms[66,67]. Due to sensory abnormalities leading to scoliosis in the majority of individuals with RS[22,65], it has been suggested[26,27] that sensory stimulation that enhances symmetrical sensations should be used as preventive measures for all individuals with RS from a young age. If executed properly, walking can enhance and preserve midline sensation; it can also be perceived as a measure for maintaining improved general medical state of the individual with RS.

- Improving blood circulation to the lower extremities — Individuals with RS present a dysfunctional autonomic system[68,69]. As a result, they show circulation problems of the lower and upper limbs. Walking improves circulation, helps muscle oxygenation, and increases metabolism of the lower extremities, thus reducing ill effects of the pathologic autonomic system of the feet (i.e., cold, edematous, bluish/red skin).
- Enhancing self-initiation and independence — One of the most common limitations of individuals with RS is their lack of initiative, with its accompanying dependency on others. Acquiring the ability to walk enables them to become less-dependent individuals with an opportunity to explore/investigate their surroundings as they wish, reaching out and discovering the world. This is an ideal situation for any person and even more so for individuals with RS. For this reason, individuals with RS should be continuously trained for walking, preferably achieving unassisted ambulation.
- Social acceptance — The manner in which a person gets about, by walking or in a wheelchair, influences the manner by which society views him/her. An individual who can walk and interact with others will have an advantage over a person confined to a wheelchair. If walking is not achieved, the individual with RS is dependent on others for initiating, approaching, or creating positive social situations thereby reducing her chances for social acceptance.
- Walking can be achieved at any age — According to parental reports on the RettNet, there are individuals with RS who have started walking at ages 3, 7, and even 18[70,71,72]. There are also reports of individuals with RS reacquiring walking ability after years of using a wheelchair[26,46,73,74]. One case study recounted a woman with RS who lost her ability to walk at age 20, but regained her ability at age 43, more than 20 years later[74].

The optimal type of walking is, of course, independent walking, free from the help of others as well as any mobility aids. Since some individuals with RS are in need of support at some stage, this chapter will also describe various types of walkers. If the person with RS is able to walk using this type of support, she should be allowed the freedom to walk independently using a walker. Another tool that the author has used successfully for training individuals with RS to walk is a treadmill (see Picture 21), with extremely positive results regarding function and aerobic conditions[53]. When properly placed, walking on a treadmill demands a minimum amount of supervision. The treadmill can be placed opposite the TV in order to increase motivation of the user and elongate exercise duration.

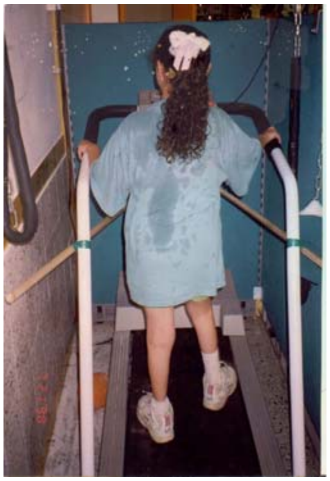

**PICTURE 21**. A child with RS on the treadmill.

## Walking Equipment

It should be emphasized that this article does not support the use of waking equipment, but if taking the social, functional, and physiological aspects into consideration, any type of mobility is preferable to sitting around leading a sedentary lifestyle. Therefore, if this is the only way to achieve mobility for the individual with RS, then adapting an appropriate walker should be considered.

Canes and crutches are most useful when the gait problem is unilateral and/or mild, but people who need bilateral canes may find that a walker works just as well and may even be easier to use[1]. There are several gait patterns that can be used with canes and crutches (e.g., swing through, opposition), some of which may be difficult to learn, especially for those with apraxia and ataxia (such as individuals with RS). If an incorrect gait pattern is used, the patient is at increased risk of falling, and the crutches may not provide the correct support[1]. Therefore, due to the manual abilities and complex motor control associated with the use of these devices, they have been left outside the scope of the present article and the use of walkers will be discussed as potential aids for the gait of the person with RS. Walkers can be used for two purposes:

1. As part of a rehabilitation program wherein the user is recovering from an injury or operation.
2. As a long-term aid for mobility when the user has a permanent difficulty with walking. This article will address mostly the latter with special consideration for users with RS.

When rehabilitation is the aim of using a walking aid, then we anticipate the process to produce gradual progress towards independent and unassisted walking. Such a process may commence with the use of a walking frame in order to enhance the user's confidence and to enable her to gain experience. About 60% of individuals with RS achieve independent ambulation[2], but some may be in need of walking aids, while some may require such accessories at later ages in case of deterioration in ambulation abilities (stage IV). Due to the effect that RS-attributed disabilities have on the individual's balance and coordination[22], mobility equipment may be required for long-term use.

A walker with no wheels at all (a four-point or pick-up walker) requires more energy and arm strength to use than the front-wheeled walker and is more complicated to use correctly[1], yet it offers little additional stability over a two-wheeled walker[76]. Due to the poor manual abilities and disparxic condition of the individual with RS, the use of walkers (with no wheels) requires the performance of sequential tasks (i.e., holding, lifting, and moving the walker forward, then advancing the legs, but not too much), which can be too difficult to master. Therefore, these appliances will be left outside the scope of the present article.

It is common knowledge that walking frames are sometimes found less practical for use on all terrains and for dealing with daily situations since they are difficult to maneuver in small spaces and cannot be used on a flight of stairs, thereby reducing the user's independence. Nevertheless, despite the fact that crutches supply a higher level of mobility, they are mostly too difficult to master by the individual with RS and will also not be considered under the scope of the present article. Walking equipment may perform one or more functions including:

- Providing greater stability and balance by extending a wider base of support
- Facilitating the walking pattern of the user in terms of speed and evenness of stride
- Improving the user's upright body posture
- Increasing the confidence of the user in his/her walking ability

## Safe Use of Walking Equipment

Walking equipment should improve mobility, however, if an inappropriate walking device is used, if incorrect techniques are adopted, or if the device is not suitable for a particular environment, the independence and safety of the user will be jeopardized. Factors that should be looked at to minimize the risk of falling while using a walking device include: arranging the home environment (loose rugs, irregular terrain, and a cluttered floor area are all potential hazards), avoiding wet floors (walking equipment should not be used in wet floor areas), as well as wearing appropriate and supportive footwear.

### General Consideration Regarding the Use of Rollators (Wheeled Walkers)

The following factors should be considered when choosing a walker:

- Research that examined several different types of walkers[77] has shown that the most important factors for adapting a walker to a person’s needs are the user’s clinical condition and home environment, rather than the conditions of the institution or educational facility.
- If a walker must be carried up and down a flight of stairs, it will soon be abandoned. If the person with RS's daily activities and physical environment necessitates the use of stairs at home or in the institution or educational facility, then two rollators should be provided to use on different floors.
- Rollators usually occupy a lot of space and building larger rooms is not always financially or spatially possible. Therefore, handles should be attached to the walls to support the user in small spaces, such as bathrooms, where the use of the rollator is excluded.
- A folding rollator should be chosen if the person travels daily by car or if her home is small and there is no place to store the rollator. (The rollators that take up the least amount of space are V-shaped walkers. These are also the easiest to maneuver, but are less stable.)
- Folding rollators must be checked when opened to ensure that they are locked so that they will not suddenly fold up when being used.
- In general cases of ataxia (other than RS), a reciprocal walker with a center axis (each side is moved alternately with the other) is sometimes recommended. It is difficult to control this type of walker and, for this reason, it does not suit the needs of individuals with RS.
- When considering the physical environment of the potential user, and whether or not the rollator is to be used in the bathroom, it is suggested to order a rollator without a high anterior bar in order to enable access to the toilet.
- The height of a walker should be carefully taken into consideration. If the frame is too high, the person will find it difficult to straighten her elbows sufficiently and will not receive enough body weight through the arms. On the other hand, if the frame is too low, it will encourage the person to bend over, resulting in poor posture. It is usually appropriate that the user’s elbows are flexed at about 15 degrees when her hands are placed on the handles. In the case of a forearm-walking frame, the height of support should bring the elbows to a 90-degree angle[75].
- The height of the frame is an important element that can be altered for therapeutic needs. The physiotherapist may deliberately set up a frame at a "wrong" height. If the user with RS is kyphotic, she can be brought to a slightly more erect position by using elbow splints or by raising the height of the walker. For individuals with RS who tend to fall backwards, on the other hand, lowering the height of the walker will force them to keep their back slanting forward, thus improving their stability. If a person with RS tends to fall backwards, walking with the back slanting forward also helps to attain a corrective sensual feeling that will enable her to remain erect in the long run.
- The user should always be measured for the height of her walking frame when wearing appropriate and supportive footwear.
- The individual with poor trunk control should be given a rollator that provides enough stability. On the other hand, since initiative of the user with RS is commonly reduced, the best rollator to consider would be the one enabling maximum maneuverability. Therefore a delicate decision making process should be performed to evaluate the suitable type of walker for each user with RS in accordance to her functional abilities and attitude.

- Due to dyspraxia, the ability of those with RS to adjust properly to difficulties within their walking path may be limited. Therefore, it must be ensured that the walking area is flat and free of gaps, steps, or other obstacles, at least during initial trials with the equipment. Regardless of all the above-mentioned safety precautions, if there is the slightest chance that the person might fall, close supervision should be administered.
- Lightweight walkers are easier to maneuver but, at the same time, are also more liable to topple forward.
- Tipping an anterior walker forward might sometimes occur due to the user walking forward within the walking frame. If this danger becomes evident when the person with RS begins to walk, it is recommended that a mechanical limitation is placed to prevent her from tipping forwards with the walking device. Examples for proper adjustments for such a problem are the use of elbow splints and the placing of a horizontal bar at pelvis level, or the non-conventional use of a posterior walker (see picture 22)
- Since the manual abilities of the individual with RS are usually lacking, a basket can be attached to the rollator, enabling the user to carry things when she ambulates. Care should be taken not to overload the basket; otherwise, the rollator could become unstable and overturn.

In many cases, the rollator needs to be altered in order to meet the needs of the individual with RS. These alterations include the following:

- Adding elbow splints — These splints can be either flexible or stiff and will help the user to keep her elbows straight or almost straight, thus bringing her hands to the proper position on the handles of the walker.
- Adapted handles — Grasping objects and holding them for long periods of time is difficult for most individuals with RS. For this reason, grasping is an important therapeutic element. Hand rests and Velcro straps can be added to the handles of the walker to help the user grasp the walker for the prolonged duration of her transport.
- Adding a bar in front — As mentioned earlier, adding a bar at pelvis height can help in preventing the user from advancing too far forward and overturning the rollator as a result.
- Adding weights — If the person with RS uses the rollator throughout the day, it should be lightweight and foldable. If it is very heavy or large, it will impose difficulties for caregivers and will probably be abandoned. If the person with RS needs a heavy and stable walker to keep her balance, homemade or store-bought weights can be attached to the walker. The weights can be kept both at home and at the educational facility, attached at the base of the rollator when the user arrives and removed before she commutes, thereby creating a situation were a lightweight walker is being transported and heavy steady one is being used for walking.
- Broadening the base — A rollator can be made more stable while still remaining lightweight by adding side wheels similar to adding training wheels to bicycle. This will broaden the base of the walker and add stability, without adding a lot of extra weight. If side wheels are added to the rear bar of the rollator, they should be double hinged in a fashion that will allow passage in narrow doorways (no picture is available).
- Adapting the wheels — The wheels of the rollator should be made of material suitable to the type of surface/terrain the user walks on. Small rubber wheels are sufficient for even floors. Larger wheels/castors are needed for traveling over rougher surfaces, such as gravel or brick walks. Inflatable wheels should be used on extremely uneven surfaces, but chances are that an individual who can successfully maneuver herself over surfaces of this type does not need a rollator at all.

The following section presents a list of rollators ranging from simple, lightweight rollators to sophisticated ones. As mentioned before, this list will not include regular walkers (without wheels), since using these types of walkers demands a combination of abilities that are too difficult for most individuals with RS. All walkers described here have two or four wheels, and are rigid and stable.

## *Types of Rollators*

### Basic Rollators (Two-Wheeled Walkers)

Standard aluminum rollators are square and have two front wheels, which move on one plane of movement and cannot swivel. Because of this, they cannot be used on unpaved surfaces or carpeting. The aluminum construction is lightweight and suitable for individuals who move around a lot. On the other hand, this type of rollator is not particularly strong or stable, and should be recommended for users with RS with poor to moderate balance. Stainless steel rollators are more stable and durable, and can be folded for easy transport and storage. However, these rollators are as difficult to steer as those made of aluminum when used on uneven surfaces.

### Three-Wheeled Rollators

Three-wheeled walkers have greater maneuverability than four-wheeled walkers[78], but they do not offer the seating advantages of a four-wheeled walker and lack the stability of a two-wheeled walker. Three-wheeled walkers are particularly helpful for people who need more support than is provided by a cane and have to maneuver through narrow spaces, such as classrooms[1]. These rollators have the advantage of being compact and foldable for easy storage, and have maximum mobility. On the other hand, their base is quite narrow and, for that reason, they provide particularly poor stability, especially while turning. Some models are manufactured with brakes that lock as soon as weight is placed on the walker; such an option offers a certain safety advantage.

### Four-Wheeled Rollators

There are several types of four-wheeled rollators:

- Posterior rollators have four wheels: two front swiveling wheels and two non-swiveling rear wheels (ferrules). These wheels have brakes that are designed to prevent the user from stepping/falling backwards. These brakes are mostly not very efficient. The advantage of this type of rollator is that it provides stability and each step forward brings the rear bar into contact with the buttocks. This walker is usually made of aluminum and can be easily folded for storage. The open front end enables the user to take broad, “normal” steps. A helmet should be considered for protection against falling injury. It is possible to use this walker in a non-conventional way (as an anterior rollator, for example) so as to achieve better stability. A forward inclination of the user can be applied as cautionary action against falling (backwards) and as a horizontal perimeter to prevent the user from stepping out of bound (see Picture 22).

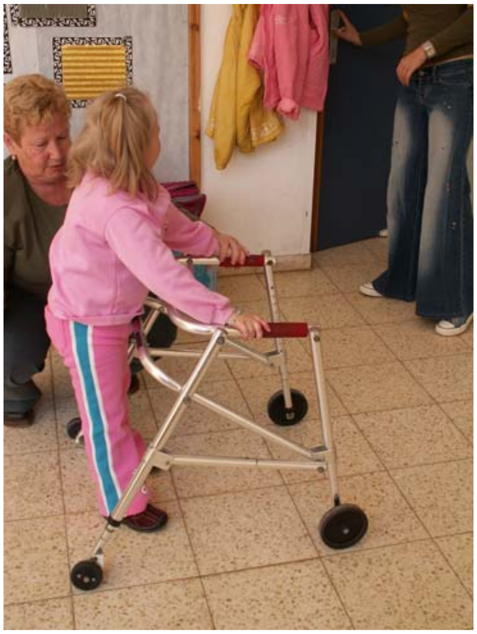

**PICTURE 22**. Non-conventional use of a posterior walker.

- Anterior (regular) rollators are extremely mobile and somewhat more stable than three-wheeled rollators. They can be fitted with a seat or basket, and are therefore suitable for daily walks around the house or for taking short walks to a nearby playground/friend. Some have weight-operated brakes. The size and type of wheels should be chosen according to the dimensions of the user and the type of surface she usually walks on. The more uneven the walking surface, the larger the wheels should be.
- Rollators with arm supports (platform armrest) are suitable for use by individuals with RS who have difficulty grasping objects. They are higher than regular rollators and are somewhat less stable, and present an increase of the overall weight and bulk of the walker[1]. Due to risk off overturn, care must be taken when moving them over doorjambs, carpets, or other obstacles. The armrest can be divided into two halves or can be used as one large area on which the individual can lean her entire weight. From my experience, this type of anterior leaning area tends to encourage the user to be more passive, while at the same time, hinders vision. A horizontal bar at pelvis height can also be added to this type of walker in order to prevent the user from moving too far forward. This offers a slight advantage of distributing weight equally on the front and rear edges of the rollators, adding greater stability.
- Rollators with trunk supports are usually made of steel and are extremely heavy, even though new plastic models are now available (see Picture 23) that are much easier to maneuver. They have four wheels, two or four of which swivel (it is best to start with all four wheels locked to increase stability and gradually, when the user gains control, to enable pivoting to all wheels). Most are designed for indoor use and are intended for individuals with poor trunk control and poor balance. Due to the support they give to the user's trunk these types of rollators seem unfit for passive individuals such as persons with RS. Yet it is possible that a few month of daily training are needed to get the person with RS to "get the hang of it" as was the case with the child presented in picture 23.

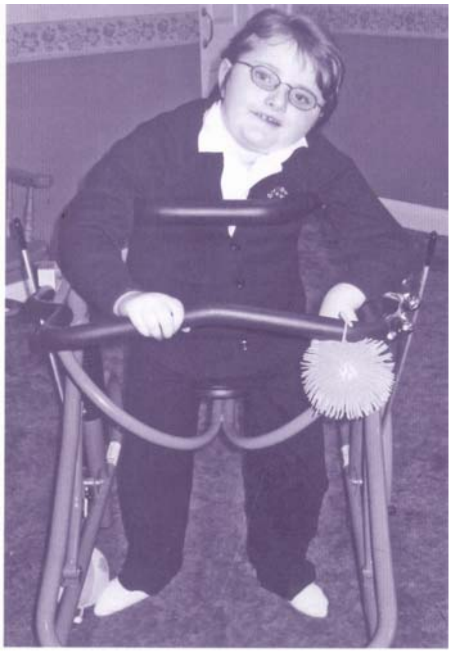

**PICTURE 23**. Use of rollator with trunk support by a child with RS.

There are three major types of rollators with trunk supports:

- Square steel frames — These rollators are equipped with removable and adjustable parts and accessories, such as front handles, a knee separator, and hip supports. The height of the rollator and of the trunk support can be adjusted. Most of these rollators have leg straps to prevent tight adduction during walking for individuals showing extreme adductor spasticity. Passive individuals are liable to sit and rest in the rollator rather than exercise; for those, alternate solutions should be considered. If the user is showing extreme flexor muscle tone, a rubber band tied to the rear poles while crossing their knees or having them tied at the ankles level can immediately bring the user to perform leg movements. These movements, in turn, cause the rollator to move forward, causing the rubber bands to stretch…..and so on and so forth (see Picture 24).
- Hart walker — The Hart walker is different from other types of rollators because it is especially designed to produce the user’s walking ability from scratch. The user is held in place inside the rollator's frame by a corset for the trunk as well as leg-support straps. The rollator has four wheels with a central bar that holds the support straps. When the user turns within the rollator, the front wheels turn with her. This type of rollator has brakes that lock automatically when the user bears weight on the walker, but unlock when she is supporting her own weight (the amount of weight that locks the brakes can be adjusted). One of the disadvantages of the Hart walker is that it takes some time to place the user in the walker and to remove her as well. This type of walker is suitable for individuals with poor trunk control, yet with motivation to walk. The

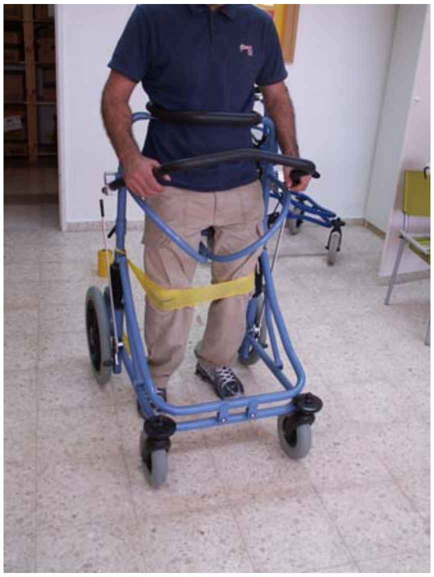

**PICTURE 24**. The use of elastic bands to enhance walking.

Hart walker has the positive advantage of forcing the user to remain active. On the other hand, a person with RS who leans on the back straps will cause the brakes to lock, and the walker will not move. Due to limits in initiative displayed by many individuals with RS, it is questionable if this type of walker is suited for the majority of this population. Nevertheless, there are some who use it[79, 80] (see Pictures 25 and 26).

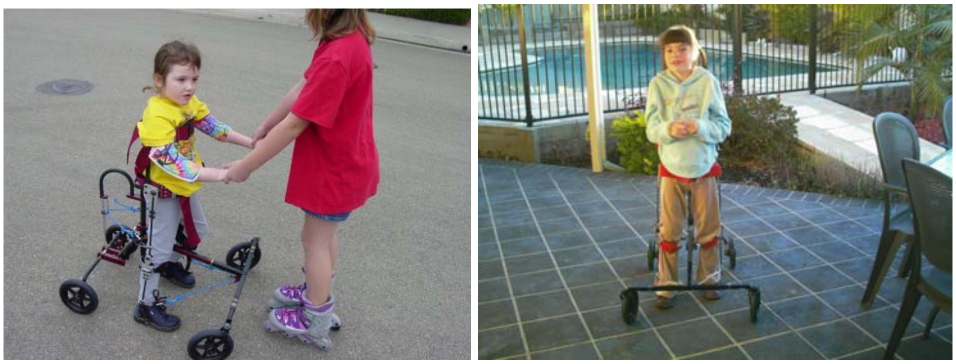

**PICTURES 25 and 26**. Children with RS using a Hart walker.

- Triangular walker — This type of walker is designed for users who cannot carry their own body weight. The base is V-shaped and usually has small wheels. There is usually a central vertical support bar for the chest as well as a seat. The user sits on the seat with her body strapped in and pushes forward using small movements of her feet. This walker offers a maximum amount of support and passive individuals will not move this type of rollator unless they are presented with strong motivational incentives.

## Bicycles

Riding a bicycle is an exciting experience that provides the rider feelings of elation, familiarity with speed, independence, and mobility[81]. Such feelings and experiences are particularly meaningful for people lacking the ability to self-ambulate. In addition to the above, using a bicycle effectively constitutes an excellent form of physical training. Riding a bicycle offers a form of outdoor enjoyment, an experience seldom shared by individuals with RS that are usually handicapped due to mobility problems. This form of recreation can be combined with a family/social activity, such as a picnic or get-together with other children at the playground. Riding a bicycle can also improve hand function. Due to all of the above mentioned advantages it is highly recommended to consider the use of bikes for individuals with RS.

There are many different tricycles and bicycles on the market, so the “right” bicycles are those that meet the needs of the specific person with RS (fitting her size, as well as providing the required trunk support, adjustability, and appropriate foot rests), caregivers, and parents (price, size, weight).

Individuals with RS who experience intolerance to movement (also termed “gravitational insecurity”) resulting from sensory problems should assume a sitting position providing extensive trunk support. Gradual bicycle riding experience wherein the user is well supported might actually help to reduce the person with RS’s fear of movement. Consulting with an occupational therapist regarding this matter is recommended.

Persons with RS can learn to ride a bicycle, acquiring a skill that can vary their daily experiences while improving their physical fitness in the meantime. When deciding to take on this task, it is recommended to begin gradually by first letting the person with RS experience the joy of other children using a bike, by showing her bicycles of different types, parked and ridden alike. Initial riding should be practiced in a quiet place, free of excessive stimulation. The person with RS may then be helped to mount the bicycle (at her own pace!), remaining seated when it is stationary (a tricycle is preferable for maximal stability thereby establishing a sense of security), and then gradually begin moving.

In many types of bicycles, the pedals can be rotated both forward as well as backwards. During the first stages of learning, it is best if the pedals can only rotate uni-directionally (some bicycles have this feature built in). Another possibility is to have the axle of the rear wheel fixed with this trait at a bicycle repair shop.

It is recommended to begin riding with a tricycle equipped with good trunk support, a seat belt, and pedals that can be strapped around the users’s feet. At first, for the sake of building a sense of confidence by the user in the new activity, it is wise to set the handlebar in such a way that allows the bicycle to move only in a straight line (some tricycles have this option built in). Gloves with Velcro strips can be used to help the user hold onto the handlebars. Another possibility that is not highly recommended is to eliminate use of the handlebars and to attach a rod to the hand bar for pulling and external steering of the bike (some beginner bikes are manufactured with this option, see Picture 27). Using this approach provides the user with the joy of movement, yet it possesses the risk that the individual with RS will soon learn that she can allow herself to be passive, exerting with but little effort. A positive aspect of such an activity is the fact that pushing the bicycle with the user’s legs attached to the pedals causes alternating movements which might, in turn, enhance reciprocal gait. Therefore such an exercise can be considered a preliminary activity presiding gait training for this population (see Picture 27).

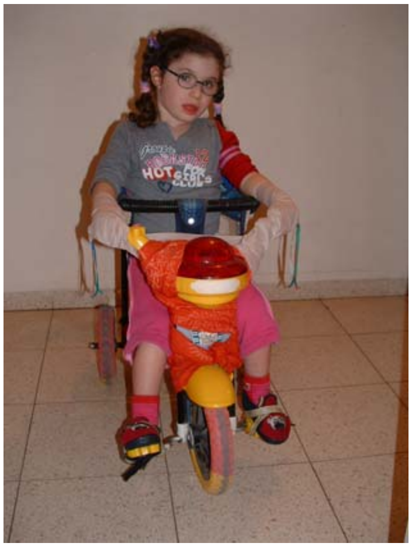

**PICTURE 27**. Using the bike as a sensory experience.

During the first stages, it is important to see that the person's trunk is well supported in order to enhance the individual's sense of security. If she enjoyed riding and would like to go again, these supports can gradually be reduced. As she becomes more and more secure, active seating should be perused in order to improve equilibrium reactions.

Songs about riding a bicycle can add motivation and provide rhythm, as well as relax the individual with RS, in case the motion of the bicycle makes her anxious. It is best to begin riding in a place with a slight incline, while going downhill the lightest touch on the pedals will move the bicycle and she will gain immediate satisfaction and reward for her efforts.

Individuals who are especially sociable, such as most persons with RS, can begin learning in a group or with a friend, since the company another person riding with her or around her, can add motivation. It goes without saying that the user should always wear a safety helmet when riding.

There are several types of bicycles that provide a solution for individuals with severe limitations who find it hard to ambulate or lack the sufficient control over their body and limbs to ride a bicycle. This type of bicycle enables the individual with RS to enjoy the exhilarating feeling of riding outdoors as well as participating in family outings and leisure activities.

- Duet wheelchair tricycles have two wheels in front and one in back, a rear seat for the adult who operates and steers the bike, and a seat for the individual with RS situated in front. The individual with RS can experience the speed, the wind on her face, enjoy the view, and participate in activities with family or friends (see Picture 28).
- Side-by-side tricycles have a wide seat with a backrest on which two people can sit side-by-side. Seat belts can be added for safety. In this sort of bicycle, trunk support by the user is required (see Picture 29).
- Bicycles with wheelchair attachments have a mechanism for easy attachment of the wheelchair to the side of the bicycle's rear wheel or to a platform, and enable individuals who lack the ability of actively riding a bike to enjoy the outdoors (No picture was available).

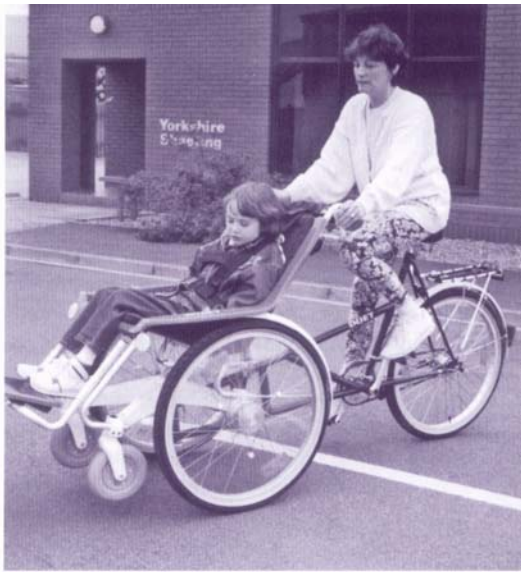

**PICTURE 28.** Duet wheelchair tricycles.

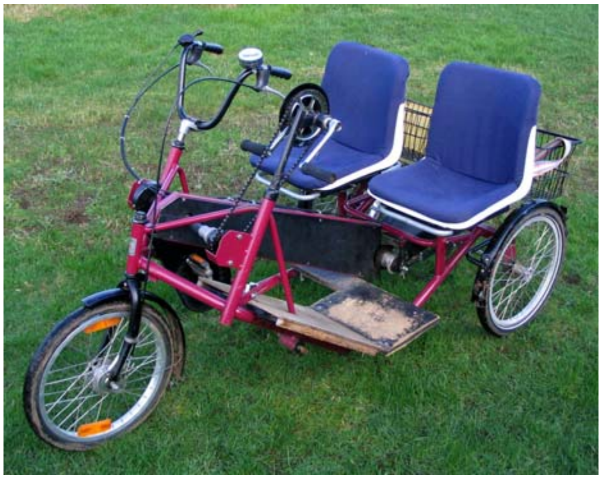

**PICTURE 29.** Side-by-side tricycles.

- Tandems and add-on trailer bikes:
  - Tandems are long bicycles with two or more seats behind each other. These bicycles allow people with multiple disabilities to enjoy the sensation of free riding. Leg motion can be obtained by strapping the user's feet to the pedals. Tandems can also be used in preparation for riding independently, or for individuals who have difficulty using their hands and keeping balance. A trunk rest and seatbelts can be added for users who have difficulty keeping their balance, and the user's hands can be strapped to the handlebars with Velcro straps to help her hold on. This type of bike can be purchased in a more conventional; two wheels model, or an adjusted three wheel version (see Picture 30)

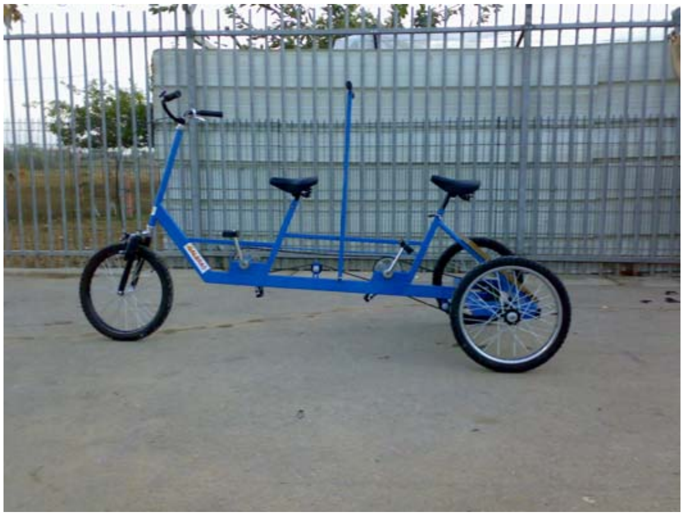

**PICTURE 30.** Tandems bikes.

- Victorian tandems (double-seated tricycles) are more stable than tandem bicycles and can be ridden more slowly. The person with RS can be left sitting on the tricycle even when it is not in motion, and the tricycle can be ridden at slow speeds suitable for all. These tricycles can be pedaled from either seat, and the front seat can be fitted with trunk support and footrests. The main advantage when using this type of bike, is that person in the back can speak to the front rider with RS and address her while they are riding together (no picture was available).

- Add-on trailer is another option that enables the individual with RS to participate in family activities and enjoy the open doors and the feeling of movement even if she does not partake in the actual action of riding. This setup can be used for familiarizing a person with RS with riding\movement in general, and as an initial step towards initiation of riding (see Picture 31).

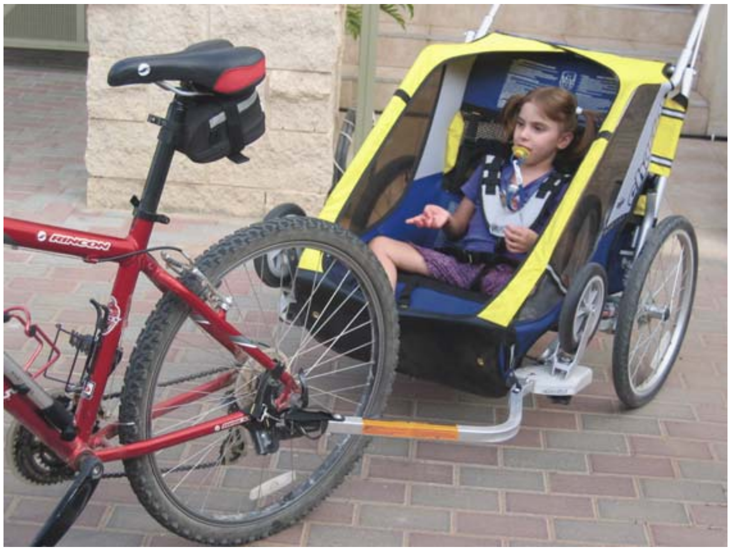

**PICTURE 31.** Add-on trailer.

- Bicycles for self propulsion - Practicing the act of getting on and off the bicycle can serve as a functional practice helping with other tasks, such as getting in and out of bed or the bathtub. When considering self propulsion one should bear in mind that most individuals with RS can control either the handlebars or pedals alone, but, in most cases cannot control both simultaneously. Some users might be more successful in operating tricycles with pedals attached to the front wheel, thereby enabling the tricycles to be moved as well as steered by the legs. On the other hand, when using this type of bike, the user's posture demands movement of the thighs and knees in a manner different to regular walking (due to the flexed position), and is therefore might be found difficult to master by the apraxic person. Some of these bicycles are foldable, enabling them to be easily transported from one place to the other without the need of a special car. A control bar can be attached to these tricycles so that the parent\caregiver can help propel or steer, in order to avoid obstacles. This type of tricycle is the most highly recommended since it offers the person with RS a higher level of independence, while enhancing respiratory and cardiovascular endurance as well as muscle activation. Individuals with RS who are usually inactive and present minor heart irregularities, shallow breathing, and inactive muscles will benefit from many aspects of riding or even pedaling a bicycle while the bicycle is controlled by an adult.
- Hand-operated bicycles are the least suitable type of bicycle for independent riding for individuals with RS, but nonetheless pose an option for using an outing as an opportunity for improving trunk and upper extremities joint ROM (Range Of Motion). This type of bicycle is designed for persons who have difficulty using their legs, but show functional manual abilities such as those of individuals with spastic diplegia type cerebral palsy. They are rigorously built, are stable, and are very safe for usage. Since these bicycles have an elongated pedal bar at chest level, the user's hands can be attached to the pedals and can then propel the bicycle, or in the case of RS, vice versa (the bicycle movement will move the user with RS's hands). An inactive individual with RS who has high muscle tone, is occupied with stereotypic movements during most of her waking hours and maintained a kyphotic posture, is in danger of losing ROM of the trunk, shoulders, elbows, and wrists. Riding this type of bicycle with the user's hands attached to the pedals enables her to attain good movement of the upper limbs and trunk, which will keep her back and arms in a more flexible condition, while at the same time participating in active leisure activity (see Picture 32).

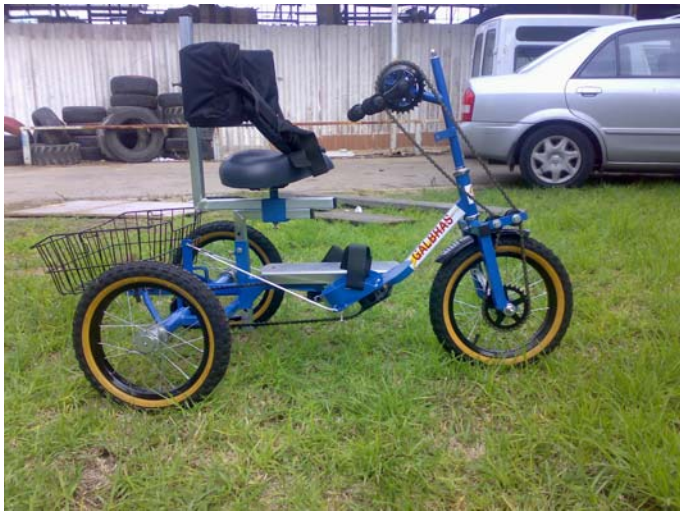

**PICTURE 32.** Hand-operated bike.

## SPLINTS AND ORTHOPEDIC SUPPORTS[82]

The decision to use an orthopedic aid and the choice of which type to use must be based on an evaluation made by a physiotherapist, an orthotist, and an orthopedic specialist. The factors influencing this decision include: leg-joint ROM, foot position, the amount of external control required by the client to enable function, the mobility of the user, her functional level, and the degree of spasticity. Since the foot and leg must also provide movement as well as stability, the influence of an orthopedic aid on these two components must be taken into consideration.

If the ankle or foot presents deviations that prevent the attainment of a neutral position when the knee is straightened without bearing weight, an orthopedic aid should not be used. Wearing an orthopedic device over a contracture at the base of the leg (the ankle or foot) will force the person to compensate in other areas, and is bound to cause excessive motion and stress to other joints as well as reduced mobility at the fixated area, an undesirable result that might cause more damage in the long run if not taken in to consideration and supported by supplementary therapeutic intervention.

It is important to emphasize that preserving the proper length of the gastrocnemius (the calf muscle) in individuals with RS is not an impossible task. The young child should be evaluated every few months and night splints should be prepared as soon as the first spastic signs appear. This might help prevent shortening of the muscles. If shortening does develop, conventional methods such as standing on a wedge (see Picture 15), passive stretching, serial casting, splints, active splints (see picture 33), or Botox injections can be used before introducing the surgical option. A daily standing program in a standing frame on a wedge has been suggested earlier in this article as means to prevent or delay muscle shortening.

If a program for passive stretching is administered by caregivers, parents, or other family members, it should be given under the guidance of a physiotherapist who will direct the manual performance of participants, supervise the administering and intensity of the program, and monitor the results.

The most efficient method of serial casting involves the changing of the cast six times on a weekly basis. This method is difficult to carry out and most programs suffice with the changing of the cast three times, once every 2 weeks. During the casting procedure, the physical therapist must ensure that the subtallar joint is in neutral position. The program must be continued even after the desired range in the ankle joint has been achieved in order to preserve the length of the muscle. When an intensive muscle elongation program is administered, resting splints should be made immediately at the end of the program by an orthotist in order to preserve the results, and postpone or eliminate the need for more intensive/aggressive (surgical) treatment methods.

Unsuccessful serial casting is usually an indication that a more aggressive plan, such as a series of Botox injections, is needed. These injections contain a toxin known as Botulinium, which causes localized paralysis of the spastic muscles. This partial paralysis enables relaxation of the muscles for a period of 3–6 months. During this period, the caregivers of the individual with RS and her physiotherapist must employ an intensive program for the lengthening of the shortened muscle. If Botox treatment fails, surgical correction is necessary. Walking and standing can help to prevent or postpone surgical intervention.

Splints and foot supports have several objectives:

- Preventing deformations — Since age-appropriate bone growth of the foot and leg is mainly achieved before 10 years of age, exerting pressure in the proper directions during those years will direct proper growth and ensure that the soft tissues are activated in the proper directions. Hypotonic children have a tendency towards deviations of the foot, such as inversion and sunken arches. The more spastic the child, on the other hand, will be liable to develop an equine-type deformation in which the foot points downwards and inwards with the toes bent. An orthotic/therapeutic solution must be implemented for each of these problems.
- Controlling and preventing undesirable movement — A splint or foot support brings the foot or ankle to a neutral position and keeps the foot within a correct ROM. It also prevents the development of shortened foot muscles as well as keeps the foot joints, bones, and tendons moving at a correct angle of motion, thereby preventing future pain and loss of function.

- Protecting weak stabilizing muscles — Muscles that are hypotonic or weak due to tendon-lengthening surgery are not strong enough to stabilize the foot when it is moving, particularly when bearing weight. Splints can provide the necessary stability and safety. Both hypotonic and spastic individuals are liable to develop abnormal foot and ankle positions. The damage caused to the foot is increased by carrying weight. For this reason, in cases where abnormal posture of the feet is caused during weight bearing, splints are indispensable for standing.
- Controlling deviations caused by abnormal muscle tone — Muscles presenting abnormal muscle tone will function through improper direction and with improper uncontrolled amount of strength. The splint prevents incorrect movement and keeps hypertonic muscles from forcing their destructive strength on the developing foot.
- Receiving correct sensory information — If the user can walk and/or stand, she will be able to try and assume positions other than sitting. Practicing standing or walking with splints offers the brain the important opportunity to receive correct proprioceptive information based on normal foot positioning.

The following list contains the most common types of foot supports:

- Semi-stiff or stiff foot supports (SPS/MBO) — These foot supports are low and preserve the position of the calcaneus, slightly stabilize minute uncontrolled movements caused by hypotonicity, help in supporting loose tendons, and prevent the drop of the medial arch, which is a common condition among young individuals with low muscle tone, which is the case in about 60% of children and 30% of adults with RS[33]. The support that this orthotic apparatus supplies, is minute and only effective when used inside a high supportive leather shoe. Such foot supports can improve stability and increase function for individuals with RS who show low muscle tone, thus helping them walk and stand with adequate foot position. This type of orthotic is ineffective if the person is extremely hypotonic, very heavy, or presents high muscle tone.
- Supramaleolar orthosis (SMO) — This type of support prevents pronation and supination of the foot and maintains dorsi and plantar flexion of the ankle in natural alignment. This type of splint is a bit more stable and offers better support than SPS/MBO. It stabilizes the foot and prevents deviation of ankle movement from the center line. Overweight individuals may need to use an SMO if lesser foot supports prove insufficient.
- Ankle foot orthosis (AFO) — This type of splint prevents plantar flexion of the foot and the stretching of the Achilles tendon, neutralizes tonal reflexes, preserves the weak gastrocnemius muscle following tendons-lengthening surgery, and can be used with additional padding as a resting/night splint. It can also be used to help stabilize the feet and ankles of individuals with RS who cannot stand independently as well as help them to maintain a standing position providing a natural articular alignment. These types of splints, used constantly under a daily/night program, can prevent the shortening of the Achilles tendon. It is important to take action before the actual shortening is noticed and apply a daily maintenance program as soon as spasticity at shin level is detected due to efficacy of early intervention. Sometimes individuals with RS might simultaneously present several deviations of the foot. One such deviation can be the shortening of the Achilles tendon together with supination and forefoot adduction. An AFO is the only type of splint that can handle situations in which a combination of several deviations simultaneously occurs. An AFO with an ankle hinge is preferable for preserving the neutral motion of the ankle during function. Hinged AFO splints are beneficial and maintain almost natural movement when the user stands up from a sitting position, as well as when she ascends or descends stairs. It is preferable to use hinged splints for mobile individuals in order to prevent loosing mobility and avoid the weakening of the triceps-sura muscles. A hinged AFO with a stop will prevent the user from exhibiting a plantar flexion posture at the ankle as well as performing knee hyperextension[24]. Since individuals with RS have difficulty adjusting to new situations and experiences, they may have difficulty getting used to splinting and hindered movement. It is therefore advisable to initially try using an "off the shelf" modular AFO before measuring and purchasing an individually custom-made one. In this manner, an assessment of how the person with RS moves with the splint, the way she has adjusted to the new sensory experience, and whether or not there are functional improvements can be observed without excessive costs.

- Active AFO — This is a long AFO, positioned over the foot, ankle, and knee joints. It is equipped with a hinge at ankle level and rubber straps in front from the top end of the splint to the anterior part of the foot. The back part of this splint straightens the knee while the Velcro straps keep the foot in place. At the same time, the rubber straps apply constant stretch over the Achilles tendon. This type of splint improves the dorsi-flexion ROM of the ankle, lengthens the triceps-sura muscles and tendons, and may eliminate the need for tendo-Achilles lengthening surgery. This type of splint should be used as a resting splint in order to maintain proper length of the triceps sura, as well as to lengthen an already shortened tendon (see Picture 33).

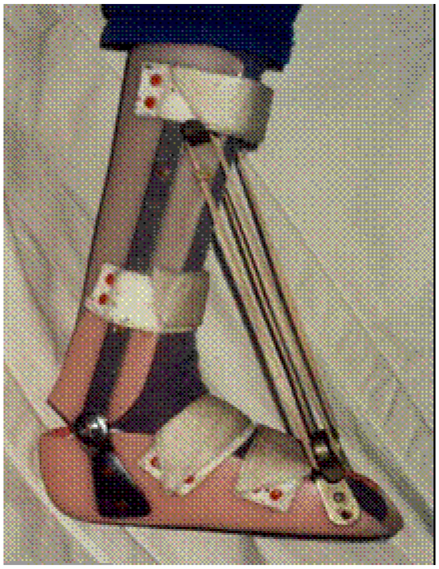

**PICTURE 33.** Active AFO.

- Inhibitive splints or bivalved leg cast — The main objective of inhibitive splints is to decrease the influence of abnormal tonal reflexes of lower extremities. (It should be noted that extreme abnormal tonal reflexes are rare in individuals with RS.) These casts are made of plaster or fiberglass. The splint can be taken apart and has front and back sections that are joined together with Velcro straps or buckles. Splints of this type must provide the following:
  - Over straightening of the large toes
  - Support and pressure application beneath the metatarsals
  - Stable positioning of the ankle
  - Deep pressure on the Achilles tendon

The sole of these splints is made flat in order to enable and encourage standing. The front part of these splints can be rounded at the front edge to help transfer weight forward and to encourage walking and moving. This splint helps to reduce muscle tone, straightens the big toes and their flexor tendons, and assists in walking for individuals who show muscle weakness under their extension hypertonic muscles which might otherwise (without the use of such supports) cause their knees to collapse.

There is a large variety of splints and foot supports available, and it is therefore important to consult with a professional before measuring, purchasing, or adapting an orthopedic aid. Individuals who are small in stature may be good candidates for devices made of relatively soft material such as Aquaplast, although it is preferable to use stiff plastic material for adults.

### Bracing

Braces are often referred to in the medical field as orthoses. They are preventive devices. They provide support or prevent deformity in cases when weakness is present. They do this by stretching or counteracting muscles that are acting abnormally. Unfortunately, they can never correct a deformity beyond the point to which it has developed. Any attempt to do so would cause pain or pressure sores. If a deformity is too great, it might not be possible to brace.

Some parts of the body are more easily braced than others due to practical reasons. The knee and hip are almost impossible to brace. The spine, foot or ankle, and the hand are the most appropriate areas to brace. Bracing for spinal deformity can make it easier to sit and, in some cases, can prevent curves from worsening. Some curves will worsen anyway, therefore the use of braces in RS should be on an individualized, trial-and-error approach. Braces for the foot and ankle may help users to keep the foot flat, and therefore stand or walk better. Any person wearing a brace, especially for the first time, should have the skin checked frequently to ensure that no pressure problems are developing. Any time a brace is used, it should add to a user's overall function and well-being, not take away from it. A parent is in the best position to observe any changes and should let the doctor know if it is making things worse.

## CONCLUSIONS

It is reiterated that the present article is a review of available scientific literature on the topic of assistive technology incorporating the clinical knowledge of the author in the field of RS; therefore scientific generalization should be limited.

Even though individuals with RS show a vast variety of functional abilities, if the use of assistive technology can improve their function and overcome their limitations, it should be promoted.

Due to the complexity of this issue, it is mandatory to introduce highly experienced clinicians in the areas of assistive technology with individuals with RS in order to enhance a process aimed towards adapting special equipment for this population.

Lack of professional assistance may cause the purchase of inappropriate equipment[5], later leading to its disuse and abandonment.

## USEFUL WEB SITES ON ASSISTIVE TECHNOLOGY INCLUDE:

- http://www.abledata.com — Provides a listing of over 17,000 different assistive devices
- http://www.wheelchairnet.org/ — Specifically focused on wheelchairs and other assistive technology devices
- http://www.resna.org/ — Site for the Rehabilitation Engineering and Assistive Technology Society of North America
